# Biomarkers of Cardiovascular Stress: A Historical Review

**DOI:** 10.3390/jcdd13080358

**Published:** 2026-07-29

**Authors:** Harshit Khosla, Jonathan Kopel, Mostafa Abohelwa, Meenakshi Awasthi, Virginia Mohlere, Sharda P. Singh, Scott Shurmur, Mohammad Ansari, Yogesh Awasthi, Sanjay Awasthi

**Affiliations:** 1 Division of Hematology Oncology, Houston and McGovern Medical School, University of Texas Health Science Center, Houston, TX 77030, USA; virginia.m.mohlere@uth.tmc.edu; 2 Department of Neurology, MedStar Georgetown University Hospital, Washington, DC 20007, USA; jonathan.kopel@ttuhsc.edu; 3 Department of Internal Medicine, Texas Tech University Health Sciences Center, Lubbock, TX 79430, USA; mostafa.abohwela@ttuhsc.edu (M.A.); scott.shurmur@ttuhsc.edu (S.S.); 4 Department of Emergency Medicine, Houston and McGovern Medical School, University of Texas Health Science Center, Houston, TX 77030, USA; meenakshi.awasthi@uth.tmc.edu; 5 Division of Hematology and Oncology, Department of Internal Medicine, Texas Tech University Health Sciences Center, Lubbock, TX 79430, USA; sharda.singh@ttuhsc.edu; 6 Department of Cardiology, Texas Tech University Health Sciences Center, Lubbock, TX 79430, USA; mac.ansari@ttuhsc.edu; 7 Department of Molecular Biology and Immunology, University North Texas Health Science Center, Fort Worth, TX 76107, USA; yogeshcawasthi@gmail.com; 8 Department of Hematology and Oncology, Texas Tech University Health Sciences Center, Lubbock, TX 79430, USA

**Keywords:** biomarkers, cardiovascular disease, cardiovascular, inflammation

## Abstract

Internal and external stress exerted by forces such as oxidative, xenobiotic, radiant, ischemic, mechanical, metabolic, neurohormonal, infectious, immunoinflammatory, emotional and psychological increases the risk of cardiovascular morbidity and mortality. Initiation and propagation of the explosive self-amplifying chain reaction of lipid peroxidation chain reaction (LPO) generates atherogenic and inflammatory toxic reactive oxygen species that cause cardiovascular tissue lesion that are the ultimate determinants of cardiovascular disease risk (CVD-R). Because the LPO toxins, cellular stress sensors and defenses, inter- and intracellular signaling, and pathogenic lesions are closely similar among these stressors, simultaneous exposure to multiple stresses can amplify LPO to accelerate accumulation of pathogenic lesions synergistically. Numerous blood tests measure LPO-derived toxins, stress-responsive metabolism and cytokines, which are used clinically as surrogate biomarkers of CVD-R. They can predict population risks quite well, but prediction of individual patient CVD-R using multiplex biomarker panels is hampered by lack of true independence between biomarkers, lack of understanding of their relative hierarchy in disease etiology or progression, and interference from comorbid diseases or acute-phase reactions. We present this historical review of landmark studies that led to the current clinical paradigms of CVD-R prediction to provide mechanistic and clinical context that will aid the development of integrated etiological biomarkers that reflect the multiple types of stress that promote CVD-R.

## 1. Introduction

A biomarker is defined as a “characteristic that is objectively measured and evaluated as an indicator of normal biologic processes, pathogenic processes, or pharmacologic responses to a therapeutic intervention” [1]. Hence, biomarkers in clinical medicine may be diagnostic, predictive, or prognostic indicators of disease pathophysiology and some are also useful as therapeutic monitoring or even screening biomarkers. The most valuable biomarkers are those that are a direct cause of a disease or a closely associated byproduct of the etiological mechanism. The ideal biomarker is one that always (or nearly always) indicates the presence of a disease (i.e., significantly elevated troponin-I is an excellent marker for myocardial infarction) or prognosticates the risk of a disease (i.e., a homozygous mutation in the Huntington’s disease gene predicts a nearly 100% chance of developing the disease).

For coronary vascular disease (CVD), though the pathophysiology (large-vessel atherosclerosis, small-vessel insufficiency, myocyte apoptosis, conduction-system lesions, etc.) has been well documented, the molecular etiological mechanism(s) are incompletely understood. Although multiple lines of evidence from epidemiology, clinical observation, basic chemistry, biology, biochemistry, molecular biology, genetics, and imaging have demonstrated that inflammation and oxidative stress are common denominators associated with cardiovascular pathophysiology, the detailed mechanisms that give rise to these conditions or the mechanisms through which these conditions cause the pathological lesions remain to be elucidated. Chronic stress runs through all three as a unifying theme [2]. The broadest definition of stress in the biological context is a potentially damaging challenge that a cell or organism faces, which affects normal equilibrium and results in a defensive or adaptive response. Humans experience a variety of pressures, which can be generically categorized as chemical, mechanical, radiative, or psychological. A common thread among these stressors is that they all accelerate the formation of lipid hydroperoxides (LOOH) formed from polyunsaturated fatty acids (PUFAs), such as the Ω-3 and Ω-6 essential fatty acids, through the reaction with oxygen free radicals [2,3,4,5,6,7,8,9], due to the etiological involvement of oxidative stress in all factors identified by the Framingham studies as determinants of CVD-R including aging [10,11,12,13,14,15], HTN, smoking [5,6,16,17,18,19,20,21,22], insulin resistance [15,23,24,25,26,27,28,29,30], obesity [19,31,32,33,34,35,36,37,38,39,40], metabolic syndrome [41,42,43,44,45], and T2DM [19,46,47,48,49,50,51]. Inflammation and oxidative stress are mutually amplifying processes that cause injury to cardiovascular tissues and most blood biomarkers of CVD-R are biochemicals that are substrates, products, or biological regulators of inflammation and/or oxidative stress [14,19,21,45,52,53,54]. The complex interrelationships have limited the use of many of them as independent biomarkers. However, the extensive studies to characterize them have provided valuable insight into the molecular etiology of cardiovascular disease, led to novel therapeutics and yielded multiplex panels of biomarkers individualized to predict CVD-R in specific populations of asymptomatic and symptomatic individuals. Despite the clinical value of given biomarkers, knowing whether it is etiological or simply a downstream event without consequence is an important distinction because non-etiological biomarkers may not accurately predict therapeutic response and may obfuscate future studies to elucidate molecular etiology. The significant residual CVD-R, as high as 50% in most clinical trials after effective therapy for all known risk factors trials [55,56,57,58], and the lack of any CVD in many individuals with multiple risk factors, highlights the continued need for improved biomarkers. Perhaps the best example of the ultimate etiological biomarker is found in another disease, peptic ulcers; although many risk factors were known for decades, a simple and effective therapy was not devised and implemented until the demonstration through all of Koch’s postulates that *H. pylori* causes peptic ulcers. Ideally, a single etiology that could be shown to cause all (or a majority) of the abnormalities we recognize as risk factors for CVD could prove to be an ideal biomarker of CVD-R as well as a single therapeutic target for which optimal therapeutics could be designed.

## 2. Clinical Biomarkers in Cardiovascular Disease

Prognostic biomarkers used for prediction of CVD-R in asymptomatic patients are distinct from those used in symptomatic patients to diagnose, prognosticate, predict and to use for therapeutic monitoring these patients with specific cardiac diagnoses. CVD is a highly heterogeneous group of diseases, and each component of CVD-R has multiple determinants, and thus multiple biomarkers. Table 1 highlights clinical laboratory studies used in the routine settings and in the specialized research context.

Physicians rely on a composite set of clinical data from a careful history and routinely available biochemical and imaging diagnostic tests to arrive at a general clinical estimate of CVD. Beyond the history, there are physical and routine labs (CBC, CMP, U/A, HgbA1C), EKG and chest X-rays. In addition, the best known, clinically validated, and economical biomarkers for prognosis and prediction of therapeutic benefits are blood lipids. The Framingham studies began with their identification as strong determinants of risk of cardiovascular death and over the next seven decades have led to the present state of the art where lipid panels measuring triglycerides, total cholesterol, HDL, LDL, VLDL, lipoprotein particle size, constituent structural proteins, and lipid metabolizing enzymes are used for predicting CVD-R in both symptomatic and asymptomatic individuals. Despite compelling evidence for the role and molecular mechanisms for these particles, the presence or absence of dyslipidemia is far from an absolute predictor of atherosclerosis or death/morbidity from CVD [59]. Classical studies of the mechanisms that regulate volume, osmolarity, and electrolyte balance by the kidney led to the elucidation of the role of cardiac origin natriuretic peptides in cardio-renal homeostasis and their central role in the molecular etiology of congestive heart failure (CHF). Blood measurements of these peptides are currently the best biomarkers for prognosis, prediction of therapeutic benefit and therapeutic monitoring in CHF. The state-of-the-art diagnostic, prognostic and predictive biomarkers for myocardial infarctions in symptomatic patients have evolved from measuring blood levels of relatively non-specific intracellular enzymes that spill into blood to highly specific measurements of cardiomyocyte proteins. Interestingly, these are highly useful, but clearly not etiological. Coagulation proteins play a central role in the etiology of MI and can also be used in prognosis, prediction and therapeutic monitoring. Of course, MI is a consequence and the ultimate endpoint of CVD-R that is to be avoided by deploying novel pharmacological and mechanical therapeutics using increasingly specific biomarkers of CVD-R in asymptomatic persons. In the present review, we have reviewed the literature on the discovery and molecular mechanisms of key CVD-R biomarkers, their relationship to lipid peroxidation, stress and cytokine signaling, and endocytic mechanisms regulated by a glutathione-linked biological stress defense enzyme, RALBP1 (Rlip76).

## 3. Troponins

Sudden death and myocardial infarction (MI) with ensuing fatal arrhythmias, congestive heart failure and certainly sudden death are the dreaded temporal endpoints of the Framingham CVD-R prediction algorithms in the general population. The population of patients who already have signs or symptoms of these conditions present a different challenge, one that requires rapid individualized risk calculation in a clinic, emergency room or hospital based on biomarkers for diagnosis, prediction of benefit for each therapeutic choice, and prognosis of survival after some specified time. Though most useful as a diagnostic biomarker, the blood levels of the muscle protein troponin leaked from damaged cardiomyocytes can also be applied in prediction and prognostication of CVD-R in specific sets of high-risk, symptomatic populations.

Troponin is a heterotrimeric globular protein (Tn) consisting of three subunits, troponin T (TnT), troponin-I (TNI) and troponin-C (TnC) [60,61]. Tn is bound at regular intervals on the surface of each turn of the helical polymer of the fibrous protein tropomyosin (TPM) [62], which is wound around the helical F-actin filament (polymer of G-actin monomers) to form the thin filament of muscle fibrils [63]. Tropomyosin sits in the groove of the F-actin helix to prevent binding of the myosin head to actin [62]. The TnT subunit serves to attach Tn to TPM while the TnI binds actin in a conformation that prevents interactions of actin with myosin [64,65]. TnC is the calcium binding subunit that, when bound to calcium, rotates TnI away from the myosin-binding site on actin to allow binding of actin to myosin. Actin–myosin binding initiates the cycle of ATP binding, ATP hydrolysis, myosin head extension, ADP dissociation, myosin head flexion, and resultant movement of myosin fibers along the actin filament to contract the muscle. Loss of integrity of the cardiomyocyte releases Tn into the blood, with the blood levels of holoprotein and individual subunits rising, peaking, and declining in characteristic profiles that reflect the persistence, severity, and resolution of acute or subacute damage.

Beyond the history, physical, and typical EKG findings, clinical diagnosis of MI at presentation is aided by measurements of cellular enzymes spilled into blood from damaged cells. Over the past six decades, diagnostic blood tests have evolved through aspartate transaminase (serum glutamate-oxaloacetate transaminase, AKA AST or SGOT) and alanine transaminase (serum glutamate-pyruvate transaminase, AKA ALT or SGPT) [66,67,68,69], lactate dehydrogenase (LDH) [70,71,72], LDH isoenzymes LDH1 and LDH2 [73], creatine phosphokinase (CPK or CK) [74], and CPK isoenzyme MB (CK-MB) [75], to the present gold standards, troponin T and troponin-I subunit immunoassays [76]. Though still quite useful clinically, AST, ALT, and LDH assays are limited in their specificity for MI because of their expression in non-cardiac tissues and by a broad range of comorbidities such as hepatitis, tissue infarction, sepsis, malignancy, pneumonia, hemolysis, or stroke that increased their blood levels. CPK is muscle specific and it is expressed in other muscle types, while CPK-MB isoform (CKMB) expression is much more specific for cardiomyocytes, and their expression in non-cardiac muscle can occasionally yield false positives in the presence of conditions such as rhabdomyolysis, malignancy, dermatomyositis, and stroke [77,78,79,80,81,82]. Like CPK, Tn expression is limited to muscle cells, but each subunit is encoded by multiple genes; each gene encodes multiple splice variant isoforms expressed in a muscle type-specific manner [83].

The isoforms of TnC are encoded by three genes but the proteins encoded by these do not have a sufficiently muscle type-specific pattern of expression and their blood measurements have not yielded useful clinical assays for diagnosis of MI [63,84]. TnI is also encoded by three genes [85], but their differential expression and alternative splicing yields a cardiomyocyte-specific TnI (cTnI) with a unique N-terminal domain with <50% amino acid homology to other isoforms. This has been exploited to develop a highly specific immunoassay for cTnI detection in this domain [86,87]. Similarly, TnT is encoded by three genes that encode four isoforms but only one is specific for adult cardiomyocytes, also allowing the development of a highly specific and clinically useful immunoassay [88,89,90].

The TnI and TnT assays have been developed, standardized, clinically validated, and extensively deployed for clinical use, essentially becoming the gold standard biomarkers [91,92]. They can detect minute amounts of myocardial damage, often at levels that do not have specific clinical manifestations, estimate the severity or persistence of injury, serve as biomarkers that predict the necessity and outcomes of acute therapeutic interventions, and be useful in prognostication of longer-term outcomes. Baseline levels of cTnI and cTnT are determined by gender and have a biphasic age distribution, decreasing during teenage years, stable through adulthood and rising after age 60 [93,94]. cTnI release after MI is monophasic with a peak by 48 h if there is no reperfusion, and earlier if the occlusion is reversed. Because it occurs earlier than the CKMB, it is a more sensitive marker early in the symptomatic course [95].

As mentioned previously, troponin T is considered the gold standard for detecting a recent myocardial infarction [91,92,96]. Given the strong prognostic information provided by troponin T, there was interest whether bedside tests for the detection of cardiac troponin T and troponin I would provide a quicker diagnosis of cardiovascular injury and, thereby, improve cardiovascular outcomes with faster treatment interventions. For the early diagnosis of myocardial cell injury in acute coronary syndromes, bedside testing for cardiac-specific troponins is very sensitive. Negative test findings are associated with minimal risk and permit the quick and safe departure from the emergency room of patients experiencing an episode of acute chest discomfort [97]. Similar results were reported by Antman et al. [98]. With the effectiveness of these assays, a clinical trial was conducted to examine the sensitivity of four different bedside tests for troponin T levels: Abbott-Architect Troponin I, Roche High-Sensitive Troponin T, Roche Troponin I, and Siemens Troponin I Ultra) and a standard assay (Roche Troponin T). The trial showed that each of the above listed assays had excellent sensitivity for troponin T [99]. In recent years, other markers, such as fatty acid-binding protein, were found to have a similar sensitivity to troponin T; however, it was found to have higher false-positive results [100]. The sensitivity of troponin T assays has further improved with the creation of high-sensitivity troponin assays [92,101,102,103,104,105,106].

With improvements in the sensitivity of troponin T assays, several clinical studies investigated whether troponin T could stratify high- and low-risk CV patients and provide prognostic information [96,104,107]. One of the first studies to examine the use of troponins for examining the prognostic potential of troponin T in myocardial infarction was conducted by Stubbs et al. Using 460 patients admitted for chest pain within three years, the study showed that patients with elevated troponins had a rate of subsequent cardiac death, coronary revascularization, or non-fatal myocardial infarction comparable to the troponin T negative group. It was therefore suggested that troponin T helps with risk factor stratification by identifying a subgroup of individuals with unstable angina who are more likely to experience subsequent cardiac events, which persists for two years following admission [108]. In addition, troponin T levels are commonly raised in PAD patients, and they are linked to greater amputation and total death rates. A considerably higher risk is predicted by even minor cTnT increases, regardless of renal function impairment [109].

Given these observations, there was debate about the use of cardiac troponin T, serum creatine kinase MB (CK-MB) levels, or electrocardiographic abnormalities for risk stratification in patients with acute myocardial ischemia. Patients with baseline serum samples who were admitted had increased troponin T levels. Compared to patients with lower troponin T levels, these patients’ 30-day mortality rates were noticeably higher. Following the electrocardiographic category and the CK-MB level in terms of the strength of the relationship with 30-day mortality, the troponin T level was the best. The study found that troponin T levels continued to be a highly reliable indicator of 30-day mortality [110]. A similar association with troponin T levels and cardiac mortality was reported by another similar study [111]. With regard to CK-MB, patients with elevations in both CK-MB and troponins benefited most from aggressive treatment. However, there was no proof that the strategy’s composite endpoint at 30 or 180 days after admission was affected by CK-MB elevation [112].

It was later shown that continuous EKG provides additional prognostic information similar to early troponin T levels among patients with unstable coronary artery disease. Furthermore, combining both EKG and troponin T measurements was the most powerful and accurate for risk stratification in unstable coronary artery disease [113,114,115,116]. The accuracy of EKG with troponin T for myocardial infarction was also found to increase the sensitivity of both markers as more leads were added to the patient [117]. An analysis of troponin T and CRP showed that troponin T, but not CRP, was predictive of cardiac risk during the initial 72 h period, whereas CRP was an independent predictor of both cardiac risk and repeated coronary revascularization during the six-month follow-up [118]. A subsequent study by Lindahl et al. examined the relationship between troponin T and CRP. Their study showed that elevated levels of troponin T and C-reactive protein were strongly correlated with long-term risk of death from cardiovascular disease. Furthermore, the contributions of both troponin T and CRP were additive with regard to long-term cardiovascular risk and outcomes [119]. In addition to CRP, elevation of troponin T or N-terminal pro-B-type natriuretic peptide (NT-proBNP) was associated with a high mortality [104,120]. Specifically, the mortality rate after revascularization was shown to be reduced in patients who had either one or both markers raised [120]. Patients without an increase in these markers had reduced 1-year mortality without any improvement in mortality after revascularization, in contrast. In fact, a substantial rise in 1-year mortality following revascularization was noted in individuals with normal troponin T and NT-proBNP levels. A greater death rate was also associated with elevated levels of C-reactive protein, Interleukin-6, creatinine clearance, and ST-segment depression. However, mortality was decreased following revascularization even when these markers were not abnormal [104,120].

In individuals with stable ischemic heart disease and type 2 diabetes, the cardiac troponin T concentration was a reliable predictor of death from cardiovascular causes, myocardial infarction, or stroke. It was not possible to identify a subset of patients who benefited from random assignment to accelerate coronary revascularization using an abnormal troponin T value of 14 ng per liter or higher [121]. With the rise in percutaneous interventions for myocardial infarctions, there was interest whether troponin T levels may be used to assess the incidence and association of elevated cardiac troponin I after percutaneous coronary intervention. Patients with elevated troponin T levels after percutaneous intervention had a greater likelihood of worse outcomes 90 days after admission [122,123]. Another clinical trial evaluated the precision of highly sensitive troponin T/advanced coronary artery disease evaluation and conventional troponin (cTn)/traditional coronary artery disease assessment for myocardial infarction during hospitalization. When compared with conventional troponin and conventional coronary computed tomography angiography assessment, highly sensitive troponin T at the time of presentation followed by early advanced coronary computed tomography angiography examination enhances risk stratification and diagnostic accuracy for myocardial infarction [124].

With the high sensitivity of troponins for a myocardial infarction, it was hypothesized that troponin T may be used in patients with unstable coronary artery disease to identify those for whom treatment with low molecular weight heparin might be beneficial. In 644 patients with troponin T levels below and above and equal to 0.1 micrograms/L, dalteparin decreased the risk of mortality or myocardial infarction. Those with troponin T levels > or = 0.1 microgram/L experienced an increasing difference throughout the course of long-term treatment compared to the placebo and dalteparin groups. In contrast, in patients with troponin T levels 0.1 microgram/L, no favorable benefit of the long-term treatment could be shown [125]. A similar trend was found in troponin T serum levels and abciximab, an anti-GpIIb/IIIa receptor antibody sued for blocking platelet function. Specifically, it was found that troponin T serum levels identified a high-risk subgroup of patients with refractory unstable angina suitable for coronary angioplasty who will particularly benefit from abciximab [126]. In addition to anticoagulants, another clinical trial found that statins reduce troponin T levels in CV patients [127]. Statins, such as pravastatin, reduced troponin concentration by 13% and doubled the number of male patients whose troponin fell by more than 25% [127]. Subsequent analysis found that these same patients also had the lowest risk for future coronary events (1.4% over 5 years) [127]. In summary, troponins are not etiological markers but are highly specific markers of cardiovascular injury that are validated for use as diagnostic, prognostic and predictive biomarkers. They represent an endpoint for risk prediction, hopefully one that can be avoided with appropriate measures by identifying high-risk asymptomatic individuals through prognostic biomarkers.

Summary: Troponins (TnT and TnI) have evolved to become the gold standard for diagnosing myocardial infarction, with high-sensitivity assays enabling earlier detection of myocardial injury. Beyond diagnosis, troponin levels provide robust prognostic and predictive value by stratifying risk in unstable angina, predicting 30-day mortality, guiding therapeutic decisions with antiplatelet agents, anticoagulants and statins, and offering additive prognostic power when combined with EKG monitoring, CRP, and NT-proBNP. Though not etiological markers, troponins remain the most validated and clinically deployed biomarkers of cardiovascular injury.

## 4. Blood Cells as Determinants of CVDR

A cardinal feature of myocardial infarction, one that is nearly universally present as the initiating event of MI, is clot formation at the site of a ruptured atherosclerotic coronary artery plaque. This has been the impetus for studies to investigate whether a constitutional or acquired thrombophilic disorder increases CVD-R. Platelets play a key role in initiating and promoting coronary thrombus formation [128,129] as evident from the almost universal use of antiplatelet agents to reduce CVD-R and to treat and prevent MI [130]. Degranulation of platelets generates localized oxidative stress that amplifies local thrombotic predisposition through generation of oxidative stress measurable as lipid hydroperoxides and downstream eicosanoid metabolites including leukotrienes, thromboxanes, and prostaglandins [131,132,133]. These are also potent initiators and promoters of local inflammation that promote the growth of atherosclerotic plaques after each plaque-rupture event [128]. Interestingly, systematic studies of the association of single nucleotide polymorphisms associated with elevated platelet count do not indicate a significant predisposition to MI, though univariate analyses suggest a small increase in stroke risk [134]. Similarly reactive thrombocytosis, as occurs with iron deficiency due to GI bleeding, asplenia, inflammation, metastatic cancer, does not appear to increase the risk of MI significantly [135], except perhaps in rare specific circumstances where other CVD-R are also present [136]. However, elevated platelet counts due to myeloproliferative disorders such as polycythemia vera or essential thrombocythemia do impose an increased risk of arterial thrombotic disorders, including MI [137,138,139]. It is not clear that the platelet number itself poses the risk because thrombotic risk may not parallel platelet count [140]. Elevated levels of blood markers of oxidative stress are also present in patients with many types of myeloproliferative neoplasms [141,142,143], and thus whether the increased platelet numbers themselves impose the risk cannot be determined. Perhaps the increased risk is not due to platelet numbers themselves but due to intrinsic platelet abnormalities that increase degranulation, endothelial adherence, or increased inflammatory cytokines [144,145,146,147]. Elevated white blood cell count in myeloproliferative disorders may pose a greater risk than elevated platelet count [148,149,150,151]. Certainly, thrombus formation by platelets is dependent on many mechanisms that have ROS generations as a common denominator [152].

Summary: Platelets play a central role in orchestrating coronary thrombus formation and generate localized oxidative stress through degranulation, producing lipid hydroperoxides and eicosanoid metabolites that amplify thrombosis and inflammation.

## 5. Plasma Proteins as Determinants of CVDR

By the early 20th century, albumin was understood to be the primary determinant of vascular osmotic pressure [153,154,155]. It was eventually purified to homogeneity followed by elucidation of its amino acid sequence [156], the nucleotide sequence of the encoding gene [157], and its crystal structure [158]. Its multiple functions [159,160] and its role in determining CVD-R are still being defined [161,162,163]. Though it has no direct role in coagulation, it determines the pharmacological behavior of many drugs and anticoagulants used in cardiovascular disease [164,165,166,167], which may determine CVD-R and obfuscate the therapeutic efficacy of intravenous albumin to increase intravascular osmotic pressure in cardiovascular disease patients with circulatory collapse [159,168,169].

While most thrombophilic syndromes predispose primarily venous thrombosis, most carry some increased risk of arterial thromboses manifesting as myocardial infarction, stroke, or acute peripheral arterial thrombi causing ischemia of extremities or even other organs such as kidneys or intestines. The most inherited coagulopathic disorders that usually pose a small risk of increased risk of MI include hereditary deficiencies of the anticoagulant proteins antithrombin III, protein C and protein S and the activating factor V Leiden mutation. Among acquired thrombophilic disorders, antiphospholipid antibody syndrome is associated with the highest CVDR. The MINOCA syndrome, myocardial infarction without coronary artery obstruction, is associated with coronary spasm and myopericarditis [170] and occurs in persons with antiphospholipid antibody syndromes such as lupus anticoagulant, anti-cardiolipin antibodies or anti-β2-glycoprotein I antibodies which can occur in persons without or with systemic lupus erythematosus [171,172]. Persons with antiphospholipid syndromes have been shown to have increased levels of inflammatory biomarkers as well as increased lipid peroxidation products in their blood [173]. Because of the rarity of these conditions, their diagnosis only when they present with other disease-specific symptoms, and the concurrence of one or more other established cardiovascular risks, a precise estimate of the independent CVD-R imposed is not possible for coronary thrombosis but evidence for stroke predisposition is better developed [174]. However, blood levels of C-reactive proteins are elevated in many of these syndromes, especially when the patient is symptomatic.

Summary: Albumin influences CVD-R indirectly through its effects on drug pharmacology and intravascular osmotic pressure, while inherited thrombophilias (antithrombin III, protein C, protein S deficiencies, Factor V Leiden) confer modest increases in MI risk. Among acquired thrombophilic disorders, antiphospholipid antibody syndrome carries the highest CVD-R, and is accompanied by elevated inflammatory biomarkers and lipid peroxidation products.

## 6. Coagulation Factors as Determinants of CVDR

Given its central role in thrombus formation, it is not surprising that blood levels of fibrinogen, Factor I, are strongly correlated with CVDR [175]. Fibrinogen is a homodimeric fibrous protein in which each monomer consists of three peptides, A, B and G, that are encoded by genes FGA, FGB and FGGR on chromosome 4 [176]. The fibrinogen genes are constitutively expressed in hepatocytes [177,178]. The rate-limiting factor in fibrinogen synthesis is the amount of FGB protein. IL-6, a pro-inflammatory cytokine produced from monocytes, macrophages and vascular endothelial cells, promotes fibrinogen synthesis by increasing FGB, whereas other inflammatory cytokines such as IL-1 and tumor necrosis factor-alpha (TNF-alpha) reduce fibrinogen synthesis [177,178]. Consequently, fibrinogen levels rise as a consequence of inflammation or other stress just like other acute-phase reactants. Beyond coagulation, fibrinogen may affect CVDR through interactions with many proteins, such as plasminogen, factor XIII, vitronectin, and fibronectin, that are involved in mechanisms of atherogenesis [176,179]. Plasma fibrinogen levels have been shown to correlate with the prevalence of ischemic heart disease (IHD) [179,180], occurrence of coronary events, and mortality from IHD. Because plasma fibrinogen is a covariate of most CVDR factors and because it is elevated in the period following an MI, like other acute-phase reactants such as CRP and von Willebrand factor antigen [180,181,182], its value as an independent risk factor has been debated. Regardless, the magnitude of IHD risk of 2.3× estimated from population studies agrees with estimates ranging from 2 to 5× observed in cross-sectional studies [10]. Though both genders are affected, the risk conferred by fibrinogen is lower in women than men by ~2–5×. Plasma concentration of fibrinogen is higher in males than females and higher in African Americans compared with Caucasians. Fibrinogen levels have been shown to be elevated in T2DM, with oral contraceptive use, smoking, or alcohol use [180,181,182]. Interestingly, HDL cholesterol in serum is inversely correlated with fibrinogen [180,181,182].

Several molecular changes, including gene polymorphisms, alternative splicing, and post-translational alterations, may affect the plasma concentration of fibrinogen and its biochemical characteristics, and subsequently the risk of CVD. The impact of fibrinogen’s molecular changes, particularly the polymorphisms in its genes, on CVDR remains unclear due to interactions with polymophisms in CRP (CRP-rs3093068) as well as genes involved in lipid homeostasis (LDL-R, PCSK-9) [183]. Multiple types of very rare mutations, mostly near the thrombin cleavage sites, have been reported and thrombophilia has also been described, but MI has not been specifically reported. The fibrinogen A peptide gene (AαIVS4 + 1G > T mutation and Aα4841delC truncation) predisposes a thrombophilia in about 25% of individuals, but association with MI or overall CVD-R is not known [184]. FGB gene mutation A68T is a rare mutation (called fibrinogen Naples) in which a homozygous mutation has been associated with arterial thrombophilia. Another fibrinogen mutation (fibrinogen Milano II) has been associated with both venous thrombosis and peripheral arterial thrombosis, but whether either cardiovascular disease or MI are predisposed is not known [185]. High plasma levels of FGG are associated with blood clots that are extremely resistant to fibrinolysis [186,187]. Mutations in the FGG gene increased the risk of myocardial infraction, CVD, and microvascular thrombosis [186,187,188,189,190] as well as the risk of ischemic stroke and cardioembolic stroke [191,192,193,194,195,196].

In addition, regulators of fibrin formation from proteolytic cleavage of fibrinogen include the serine protease plasminogen activator inhibitor-1. After controlling all other risk factors, elevated PAI-1 plays a crucial role in platelet activation and aggregation and is also a good measure of endothelial dysfunction. It has been correlated with a 1.5-fold higher CVDR in patients with T2DM [197], suggesting that endothelial dysfunction precedes T2DM and occurs via mechanisms independent of insulin resistance, central obesity, or chronic inflammation. D-dimer levels are one of the most sensitive and specific markers of coagulopathy and their levels in the blood. In a study of 485 patients compared with 934 controls, D-dimer levels were a more significant predictor of acute coronary events than CRP or IL-6 [198]. Unfortunately, in multivariate analyses, measures of hypercoagulability have failed to significantly improve the ROC for CVD-R based on the FRS alone.

Genetic variants in prothrombin (factor II), factor V, factor VII, and factor XIII have been linked to an increased predisposition to arterial thrombosis [199]. The single basepair prothrombin mutant (G20210A) was associated with a mild venous thrombophilia and perhaps a small increase in CVD-R, likely <1.5× [200,201,202,203]. The factor V Leiden mutation (R506G) has been reported to be associated with a 1.5× risk of premature coronary disease while another polymorphism (rs1799963) was associated with a lowered CVD-R, 0.7× [204]. There may be a gender-specific effect of factor V Leiden mutation with women being preferentially affected [205]. CVD-R conferred by prothrombin G20210A and factor V R506G amplifies coexistent thrombophilic states such as hyperhomocysteinemia, elevated CRP, elevated fibrinogen, autoimmune disease, cancer, chronic disease and acute illness [206,207,208,209].

Large genome-wide association studies (GWAS) have confirmed small risks of athrosclerotic disease conferred by Factor V Leiden (1.17×) and prothrombin G20210A (1.31×) [210]. Elevated levels of factor VII protein clearly conferred a 1.79× risk of MI and low factor VII level because the -402A promoter polymorphism reduced risk by 0.6×. Cell surface glycoproteins belonging to the integrin family expressed on the surface of platelets serve as receptors for collagen, fibrinogen, von Willebrand factor, and prothrombin. The GPIb-GPV-GPIX complex binds von Willebrand factor and functions in the adhesion of platelets to vascular walls. GPIa/GPIIa and GPVI serve as collagen receptors, the latter activating expression of phospholipase C through a Src pathway. Elevated levels of the von Willebrand factor (v-WF) play a crucial role in platelet activation and aggregation and are also a good measure of endothelial dysfunction.

Summary: Fibrinogen levels strongly correlate with ischemic heart disease risk (approximately 2.3-fold), though its value as an independent risk factor is debated given its co-variance with other CVD-R factors and acute-phase reactant behavior. Mutations in fibrinogen genes—particularly FGG—increase MI and stroke risk, while PAI-1 and D-dimer serve as markers of endothelial dysfunction and coagulopathy.

## 7. Natriuretic Peptides

Blood levels of natriuretic peptides and troponin isoenzymes are the most widely used and powerful diagnostic biomarkers primarily in symptomatic persons. Natriuretic peptides are sensitive to myocyte stretch and troponins are a marker of cardiac injury [211,212]. They are used primarily to distinguish complex clinical presentations of acute coronary syndromes and the presence of congestive heart failure. In these patients, they are also powerful biomarkers of treatment response as well as short-term prognosis. They are also used as predictors of response to certain therapeutic interventions and cardiac biomarkers. Cardiac patient management guidelines recommend their use: (a) for the evaluation of cardiovascular risk in patients undergoing major cardiac or non-cardiac surgery [212,213,214], and in asymptomatic high-risk subjects of the general population aged >55 years and with comorbidities [212,215]; (b) for the evaluation of cardiotoxicity caused by administration of some chemotherapy drugs in patients with malignant tumors [212,216,217]. The measurement of natriuretic peptides (especially BNP and NT-proBNP) is recommended for diagnosis, monitoring and prognosis of all patients with heart failure [212,218,219].

The first natriuretic peptide (NP) created by stretched heart tissue to be discovered and researched in HF patients was atrial NP (ANP). It was quickly supplanted by B-type NP (BNP) and its prohormone fragment, N-terminal pro B-type natriuretic peptide (NT-proBNP), which are peptides mostly produced from the ventricles. In the treatment of patients with known or suspected HF, these 2 peptides are now the most frequently employed biomarkers [220,221]. Patients with dyspnea of unknown cause (dyspnea of unknown etiology), individuals with heart disease who are at risk for HF, and patients who appear healthy but are more likely to develop HF can all be diagnosed with HF using NPs [211,220,221]. Despite its usefulness, NP levels must be evaluated considering a patient’s characteristics, such as age and body mass, as well as laboratory tests, such as cardiac imaging, much like any other laboratory value.

Between 1978 and 2000, after and during MI in the hospital, CHF developed in 18–37%, with one-year mortality of 16–39%, and the most consistent risk factors for the development of heart failure after myocardial infarction were advanced age, female sex, diabetes, and an increased heart rate at the time of admission. Though these studies are over two decades old and thus likely do not translate directly into the present age of advanced medical technology, CHF continues to be a very frequent and pre-mortal endpoint of CVD in persons with hypertension as coronary vascular disease [222].

The discovery of ANP was rooted in studies of the pathophysiological mechanisms of edema in congestive heart failure. The kidneys were known to play a key role in the regulation of blood volume and body fluid osmotic pressure through a reflex mechanism in which increased blood volume increases renal excretion of salt and water [223,224,225,226]. Thus, reduced cardiac output due to CHF reduces renal glomerular filtration rate leading to retention of sodium and water. However, the observation that a renal diuretic can be triggered by simply shifting blood volume toward the thorax led to the postulate of an extrarenal volume sensor located in the thorax [226,227] and the subsequent identification of atrial stretch receptors that induced diuresis upon intravascular volume expansion. The effector diuretic response to increased atrial stretch was independent of vagus nerve function, antidiuretic hormone or aldosterone [228,229] and led to the postulation of a natriuretic hormone. Spherical granules are in the sarcoplasmic core of atrial muscle fibers [230,231]. The right atrial muscle was more granular than the left and the granularity decreased, consistent with their release, upon sodium or volume overloading [232]. Further characterization of these granules revealed that they contained protein [233], subsequently dubbed “atrial natriuretic factor” that was secreted in response to increased central venous pressure, resulting in diuresis [234,235]. Purification and isolation of atrial homogenates revealed that the natriuretic and diuretic activities were exerted by at least three biologically active peptides [236,237]. Structural characterization of these peptides revealed that they were highly conserved across species, contained a 17 aa membered ring due to an internal cysteine disulfide, and were of varying molecular weights due to variable proteolysis of a higher molecular weight precursor (pre-pro-atrial natriuretic peptide, pre-pro-ANP) that underwent post-transcriptional proteolysis to pro-ANP, which was further hydrolyzed into the smaller but biologically active peptides. Depending on the sequence and length of these peptides derived from cellular and extracellular proteolytic events, they exert variable diuretic, glomerular or systemic arterial vasodilator, mineralocorticoid, angiotensinergic, metabolic, cardiac antifibrotic, and even antimicrobial properties [238,239,240,241,242,243,244].

The atrial natriuretic factor/peptide (ANP) is encoded by the human NPPA gene (1p36.22) that is highly conserved among vertebrates. It encodes pre-pro-ANP, a 152 aa precursor protein [245,246] consisting of an N-terminal signal peptide, which when cleaved by an endoplasmic reticulum peptidase [247] yields the 126 aa, pro-ANP. The ANP pro-peptide is secreted into blood in response to the right atrial stretch caused by volume expansion. In addition, it is secreted at a baseline level in a circadian fashion with peak during sleep [248]. Its physiological role in vasodilation is supported by studies showing that mice lacking this gene are hypertensive. The transmembrane cardiac serine peptidase CORIN (aka atrial natriuretic peptide-converting enzyme) expressed on the surface of cardiomyocytes cleaves the N-terminal 98 aa peptide at arg-98 to yield the C-terminal 28 aa mature ANP [249]. The 17 aa disulfide loop of ANP has the motif xxxxxxxCFGxxxDRIxxxxxLCG that is identical from zebrafish to man and the adjacent residues that are not identical are structurally homologous [250]. The N-terminal peptide is further cleaved intracellularly and in the circulation to yield three peptides called long-acting natriuretic peptide (LANP, aa 1–30), vessel dilator (VD, aa 31–67), and kaliuretic peptide (KP, aa 79–98). Natriuretic peptides secreted by other tissues, such as the kidney, brain or testis, differ in size due to differences in proteolytic processing [223,251,252]. The proteolytic sensitivity of ANP to neutral endopeptidases in plasma renders its plasma half-life very short [253,254]. Volume loading activates the secretion of proANP [241]. These regions can be distinguished by differential but overlapping degrees of vasodilatory, natriuretic, kaliuretic and proteinuric effects. LANP, VD and KP lower blood pressure by dilating the aorta [255]. The natriuretic effect of ANF is of shorter duration than the other three. KP has the greatest kaliuretic effect and it can be distinguished by its ability to inhibit Na/K ATPase and cause prostaglandin E2 release [255,256,257,258,259]. All four exert proteinuric effects measurable by β2-macroglubulin levels in the urine. Infusion of full length ANP activates post-prandial lipid oxidation [260]. LANP activates cGMP synthesis and prostaglandin E2 synthesis. Potentially through effects on binding to other receptors, natriuretic peptides have been implicated in multiple other physiological functions in the CNS [261,262], GI smooth muscle [263], and cartilage or bone [264,265].

Another atrial natriuretic peptide BNP was discovered after ANP and was so named because it was first purified from brain tissue, but subsequently its expression is greatest in the heart. It is encoded by the NPPB gene, a paralog of NPPA encoded at the chromosome 1p36.22 locus. It is phylogenetically related to NPPA according to its nucleotide sequence homology; having arisen from an ancestral gene NPC-3 through a duplication event [266], its amino acid sequence is quite distinct, sharing only 32% sequence identity. Though pre-proBNP is shorter than ANP at 134 aa, it is longer than BNP (32 vs. 28 aa) but its structure contains a similar disulfide-linked 17 aa circular peptide [267]. BNP is synthesized de novo during ventricular stress, unlike ANP which is stored in aortic cardiomyocyte granules [250]. Unlike the predominantly atrial localization of ANP, BNP is expressed more in cardiac ventricles, particularly in patients with CHF. Indeed, the dynamic range of blood BNP levels is greater than that of ANP and its levels correlate with the severity of CHF. The longer plasma half-life of BNP may underlie its greater suitability for clinical use as a diagnostic biomarker of CHF [249]. While ANP appears to play a greater role in hypertension, the functions of BNP may be more directed at regulation of myocardial interstitial remodeling during CHF [268].

CNP is another human natriuretic peptide encoded by the NPPC gene at chromosomal locus 2q37.1. It appears to have diverged phylogenetically from ANP and BPP earlier during evolution. This is supported by greater sequence similarity in the AA sequence of CNO between mammals. Its aa sequence homology to ANP and BNP is relatively low (29 and 30% sequence identity with ANP and BNP, respectively). The pre-pro-CNP peptide is only 126 aa, shorter than ANP or BNP [269], and the C-terminal peptide analogous to ANP and BNP is only 22 aa; however, a longer 53 aa C-terminal peptide may be the most active form [270]. However, it also contains the 17 aa disulfide-linked loop and exhibits vasodilating and natriuretic effects. It also has distinct functions in that it exerts a broad smooth muscle relaxant activity and exerts effects on chondrocyte and osteocyte differentiation through distinct receptors [271]. Unlike ANP, it is not pre-made and stored in granules, instead being secreted in response to stress and growth factors such as TGF [272,273]. Its short half-life renders it less suitable as a biomarker [274] and the lack of hypertensive phenotype in knockout mice argues against a cardiovascular role [275,276].

The functions of atrial natriuretic peptides are mediated by intracellular signaling events activated upon binding to specific single transmembrane domain G-protein receptors NPR1 and NPR2, the former binding ANP and BNP and the latter binding CNP. NPR1 (1q21–22) undergoes ligand-independent tetramerization through disulfide linking [277] with the greatest affinity for ANP followed by BNP and very little for CNP [278]. NPR1 is subject to activation through phosphorylation by PRKCA [279]. Binding of the ligand activates cGMP synthesis in target cells [280,281,282]. Mouse knockout studies indicated that NPR1 is likely the most important mediator of the vasodilating effects of both ANP and BNP, strongly supported by the fact that the copy number of NPR1 has a dose relationship with HTN [282,283]. Though its role in human HTN can be debated [284], in the mouse, the ANP-BNP/NPR1 signaling axis has essentially the completely opposite effects of the HTN-promoting renin–angiotensin system [285] and considerable indirect evidence for its role in human HTN is provided by the association of HTN and ventricular hypertrophy with single nucleotide polymorphisms and microsatellite marker variation in the NPR1 promoter region [286,287,288]. However, NPR1 does appear to be a determinant of cardiac hypertrophy [289]. NPR2 (NPRB) is the receptor for CNP and is quite similar to NPRA in structure and function and may also be a low-affinity receptor for BNP [290]. Consistent with the role of CNP in bone and cartilage morphogenesis, mouse knockout of NPRB results in dwarfism [291] and acrosomelic dysplasia has been observed in humans with homozygous loss of function mutations [292] and short stature in those with heterozygous mutation [293]. However, knockout mice also have a hypertensive phenotype arguing for a cardiovascular function of this receptor. Indeed, it may play a greater role in CHF than NPR1, though this may be species-specific [294,295]. A third receptor, NPR3, is a decoy receptor that is unable to initiate intracellular signaling because of the lack of a guanyl cyclase domain [276], and thus functions to remove ANP, BNP and CNP from the circulation with greatest affinity for ANP and CNP and less for BNP [296]. Interestingly, it is the highest expressed receptor in the endothelium, suggesting that endothelial cells function in clearance of the NPs [297]. Its functional role as a physiological inhibitor of NP signaling is indicated by bone overgrowth as well hypotension and salt wasting in knockout mice [298].

Beyond the tissue-specific differences in the expression and sequence of the various NPs secreted by the heart and other tissues [299,300,301,302], the myriad functions of the NP signaling system [303,304,305,306,307,308,309] are determined by tissue-specific expression of the NPR1, 2 and 3 receptors [303,310]. NPR1 is not expressed in skeletal muscle and is minimally expressed in conduit vessels such as the aorta; it is richly expressed in the endothelium and smooth muscle of resistance vessels that are primary determinants of hypertension, including small arteries and arterioles. The specificity and binding affinity of these receptors to each type of NP and cell-specific differences regulate differential downstream signaling that causes cell-specific effects. Vasodilation is mediated through cGMP formation and the accompanying reduction in cAMP along with the increase in intracellular calcium [311]. The effects of ANP are largely independent of nitric oxide. Relaxation of vascular smooth muscle upon NP-mediated hyperpolarization of endothelial cells through cGMP accumulation, with consequent smooth muscle cell hyperpolarization through gap junction connections, has been proposed. This model is supported by studies showing that endothelial cell-specific knockout of the RGS2 GTPAse, the activating protein for the Gq/11α and Gi/oα subunits of NPRs, causes hypertension [312,313]. Additionally, CNP produced in the endothelial cells may activate NPRB in the neighboring vascular smooth muscle cells (VSMCs) to cause vascular dilation [273,314,315].

The link to inflammation and pro/anti-fibrotic or tissue remodeling appears to be through modulation of gene expression of pro- and anti-inflammatory cytokines such as TNFα, IFNG, IL-6, TGFB, MIF and IL10, and transcriptional regulatory mechanisms such as NFKB and cFOS subunits of the AP1 transcription factor [316]. The inflammatory cytokines, matrix metalloproteases and their inhibitors, as well as extracellular matrix proteins controlled by NFKB, may underlie sodium excretion defects as well as glomerular and cardiac muscle fibrosis in NPR1-deficient mice [317,318]. Reduction in CVD-R through NPR1 activation may also be related to the effects of elevated cGMP on the activation of PGC-1a (a coactivator or PPARG) through enhanced insulin signaling and promotion of fat oxidation due to enhanced mitochondrial biogenesis [319]. Indeed, type II diabetes has been associated with lower NP levels, defective NP receptor signaling and increased NP clearance receptor of NPR3 in human muscle [320].

Summary: Natriuretic peptides (ANP, BNP, CNP) are cardiac-origin peptides responsible for cardio-renal homeostasis and have therapeutic value, serving as biomarkers for diagnosis, prognosis, and therapeutic monitoring in heart failure. ANP and BNP signal primarily through NPR1 to mediate vasodilation via cGMP, opposing the renin–angiotensin system, while CNP acts through NPR2 with broader smooth muscle relaxant effects. The NP signaling system links to inflammation and fibrosis through modulation of cytokines (TNFα, IL-6, TGFB) and transcription factors (NFκB), and NPR1 copy number shows a dose-dependent relationship with hypertension, underscoring the system’s central role in cardiovascular pathophysiology.

## 8. MicroRNAs

The exceptionally broad and pleiotropic effects of NP signaling appear to be through regulation of NPR1 expression by certain micro RNAs that have been associated with expression of genes linked to cardiovascular disease. Many studies support the role of microRNA as potential diagnostic, prognostic, and severity markers. Mir-622, -519 and -499 are related to the etiology of CHF and miR-22-3p as severity biomarkers. Let-7i-5p, miR-223-5p, miR-423-5p, miR-21, miR-1306-5p and miR-122 are correlated with prognosis as well as survival after COVID-19 [321,322,323,324,325]. Recent experimental evidence in cultured cells transfected with miR-128 and miR-195 inhibited NPRA expression [326].

## 9. Ralbp1/Rlip76

**Figure 1:**
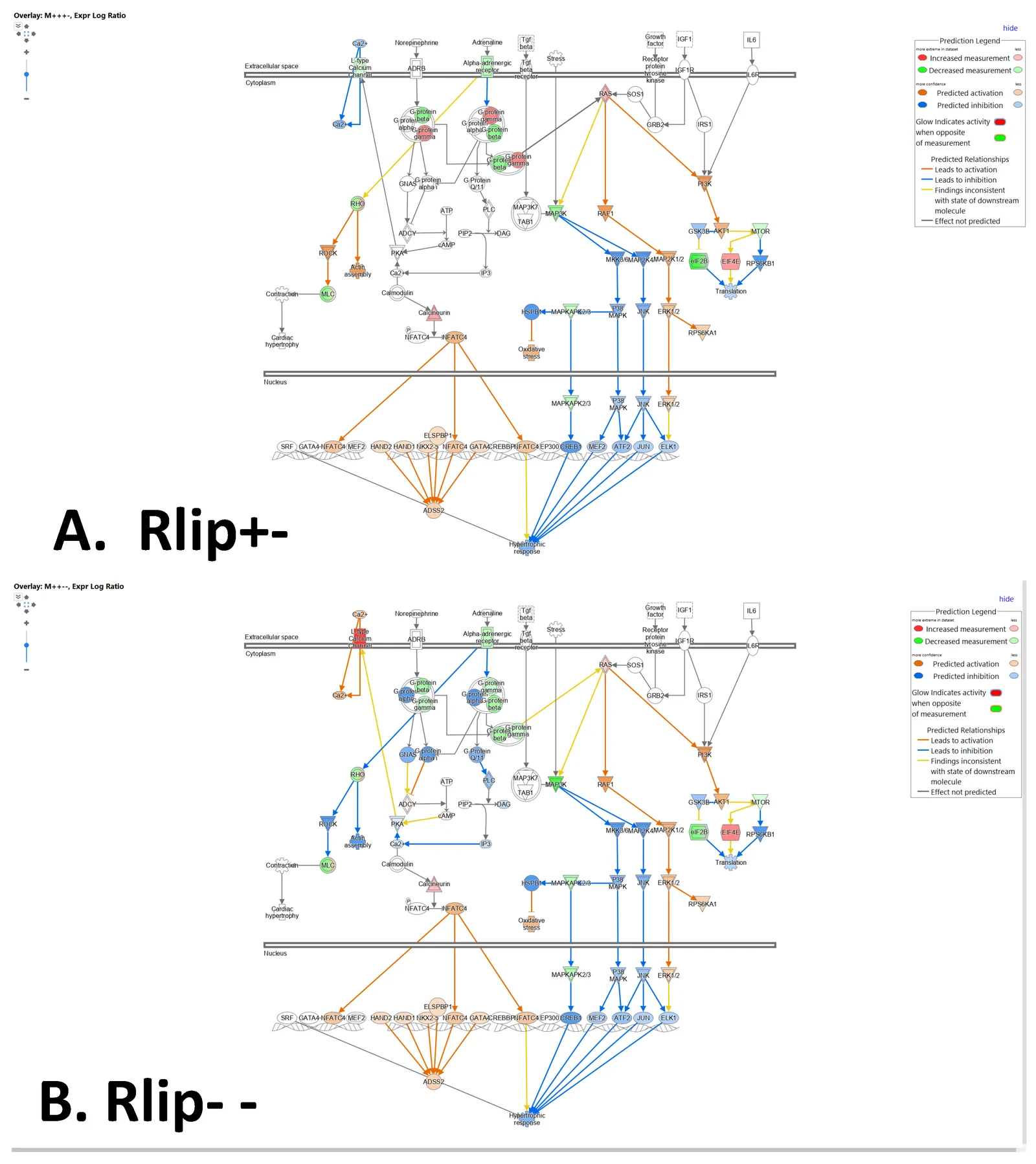
Change in cardiovascular risk pathways by presence and absence of Ralbp1/RLIP, affecting cardiomyocyte hypertrophy and function.

One of the most interesting gene networks regulated by these miRNAs linked to cardiovascular disease [327] is clathrin-dependent endocytosis (CDE) of ligand–receptor complexes. An extensive body of experimental evidence has shown that all NPR receptors are subject to CDE for their internalization and subsequent degradation by lysosomes or recycling to the membrane surface through Ral-, Rac-, and Rho-regulated intracellular vesicle trafficking pathways [328]. RALBP1 is a stress-inducible, Ral/Rac/Rho pathways-regulated nucleotidase with pleiotropic functions in stress defense. It couples its ATPase activity with efflux of glutathionylated metabolites of lipid-peroxidation byproducts such as 4 hydroxynonenal and the alpha, beta-unsaturated enols generated by peroxidation of proteins during oxidative stress and causes the internalization as well as endocytic vesicle trafficking. The rate of internalization of numerous sulfur-knot peptide signaling ligands (i.e., EGF, FGF, PDGF, WNT, etc.) is reduced by ~80% in the RALBP1 knockout mouse cells. Interestingly, RALBP1 knockout mice have an interesting metabolic syndrome phenotype directly relevant to the risk factors for cardiovascular disease including type II diabetes, metabolic syndrome, obesity, oxidative stress, glutathione metabolism, gender-linked differences in xenobiotic metabolism and malignancy (Table 2). RALBP1 knockout mice are extremely insulin sensitive, hypoglycemic at baseline, hypocholesterolemic, hypotriglyceridemic, and highly resistant to high-fat diet-induced obesity as well as to the oxidative stress caused by high-energy radiation including UV light and X-rays. This is particularly remarkable because their tissues have high levels of lipid peroxidation byproducts, substrates of the mercapturic acid pathway in which RALBP1 plays a rate-limiting role because of its necessity to remove glutathione conjugates from cells. RALBP1 loss in mice causes global loss of epigenetic remodeling mediated by promoter CpG island methylation. Though the NP pathways or the cardiovascular system have not been characterized in detail, these mice do have lower than normal cardiac weight. Recent studies to characterize these mice with respect to their transcriptomic profiles have revealed that many genes that comprise several canonical pathways directly related to cardiovascular disease including cardiovascular hypertrophy and cardiac oxidative stress signaling (Table 1) are differentially expressed. The key differentially expressed genes that unite these pathways include MYC and JUN (which form the AP1 heterodimer with FOS). For instance, signaling diagrams generated from these differential expression data (using Ingenuity Pathway Analysis software v.2020) predict that reduction in Ralbp1, as has been shown to occur with reduction in oxidative stress, should result in inhibition of the cardiac hypertrophy pathways (Figure 1).

Though the significance of RALBP1 in the mechanisms of epidermal–mesodermal transition (EMT), stem cell transcription factor and malignant transformation has been clearly demonstrated in studies showing that haploinsufficiency of RALBP can almost entirely suppress carcinogenesis due to the loss of the p53 tumor suppressor in mouse models, the significance in cardiovascular disease awaits completion of ongoing studies to fully characterize the cardiovascular structure of the knockout mice and clinical studies of RALBP1 in humans with cardiovascular disease in a manner so extensively characterized with NPs. Elevated blood levels of the Rlip splice variant protein encoded by RALBP1 in the serum of persons with metabolic syndrome compared with normal individuals suggest its potential use as an etiological CVD-R biomarker that unifies many forms of stress under a novel paradigm that links CDE with detoxification of lipid hydroperoxides, common denominators of stress. Taken together, the body on the structure and function of this protein indicates that it serves as an effector mechanism that translates stress into stress-related diseases including diabetes, obesity, and cancer.

Summary: RALBP1 is a stress-inducible protein that couples ATPase-driven efflux of glutathionylated lipid peroxidation metabolites with clathrin-dependent endocytosis of ligand–receptor complexes, linking oxidative stress detoxification to receptor signaling. RALBP1 knockout mice exhibit a striking metabolic phenotype—extreme insulin sensitivity, hypoglycemia, hypocholesterolemia, and resistance to diet-induced obesity—along with differential expression of genes in cardiac hypertrophy and oxidative stress pathways, suggesting its potential as a unifying etiological CVD-R biomarker.

## 10. C-Reactive Protein (CRP)

C-reactive protein (CRP) was first identified in the sera of patients with the acute pneumococcus infection by Tillet and Francis in 1930. Since CRP was the third component to be isolated, CRP was initially referred to as “fraction C”; however, it was later renamed to CRP to denote the unknown protein’s interaction with the capsular (C)-polysaccharide of pneumococcus pathogen [329]. Nearly ten years later, Oswald Avery and Maclyn McCarty defined CRP as an acute-phase reactant after discovering that several diseases (e.g., rheumatic fever and myocarditis) had higher concentrations of CRP in the plasma [330]. During the 1940s and 1950s, several other studies showed that CRP was a biomarker of inflammation for CVD.

Given its importance in inflammation, the location of CRP synthesis remained elusive until J. Hurlimann’s seminal study showing that CRP was primarily expressed and released from liver hepatocytes [331]. Once released from the liver, CRP was shown to have a half-life of around 18 h. Since J. Hurlimann’s initial study, reports of extrahepatic CRP production have also been observed in human atherosclerotic plaques, macrophages, and smooth muscle-like cells [332,333,334]. Within the liver, CRP is synthesized as a homopentameric protein known as native CRP (nCRP), which can irreversibly dissociate into five distinct monomers at the sites of inflammation and infection, known as monomeric CRP (mCRP) [335]. The nCRP isoform initiates phagocytosis, stimulates apoptosis, and activates the traditional complement pathway. On the other hand, mCRP can delay apoptosis and encourage chemotaxis and recruitment of circulating leukocytes to inflammatory sites. To prevent and stimulate NO generation, the nCRP and mCRP isoforms act in opposite ways. Interleukin-8 and monocyte chemoattractant protein-1 production of pro-inflammatory cytokines are increased by mCRP, although nCRP has no discernible impact on their levels. Subsequent studies have also shown that the presence or absence of Ca^2+^ can change the equilibrium between either CRP species [336,337].

The structure of CRP consists of a single type of subunit with a molecular weight of about 21,500 daltons consisting of 206 amino acids [338]. Further biochemical analysis showed that CRP belongs to the Pentraxin protein family, which consists of acute-phase proteins, such as CRP and serum amyloid P component (SAP). CRP contains five identical nonglycosylated globular subunits, each of which is made up of two β-pleated sheets that are noncovalently linked and organized in a symmetric cyclic pattern around a central pore to form pentameric, discoidal, and flattened structures [339,340,341,342]. Calcium ions are also needed for the activity of CRP. The two overlapping Ca^2+^-binding sites on each subunit allow CRP to bind two Ca^2+^ ions [343,344]. Asp60, Asn61, and several glutamate residues (Glu138, Gln139, Asp140, Glu147, and Gln150) in a loop coordinate the two Ca^2+^ in CRP. In the absence of bound Ca^2+^, this loop disengages from the CRP molecule’s main body, revealing a proteolytic site that would otherwise be buried. As such, bound Ca^2+^ are also essential components of CRP’s structural makeup and guard it against proteolytic cleavage. Furthermore, there is only one intramolecular disulfide bond present between each subunit within CRP, which is affected by temperature, pH, and protein amino acid content. CRP molecules may clump together in environments with a lower pH or more protein. In highly alkaline solutions or after several freeze–thaw cycles, CRP may dissociate into subunits or intermediate forms. CRP’s primary Ca^2+^-dependent ligand is phosphocholine (PCh) [345,346,347,348]. All eukaryotes and many prokaryotes possess the PCh-containing compounds that CRP binds to, which include pneumococcal C-polysaccharide, injured cells, necrotic cells, apoptotic cells, altered lipid bilayers, and pulmonary surfactant lipids. CRP also binds to phosphoethanolamine and other phosphate monoesters in a Ca^2+^-dependent manner. Each CRP subunit possesses a PCh-binding site close to the Ca^2+^ binding sites. All five PCh-binding sites on the CRP pentamer are located on the same face because the subunits in the completed pentamer have the same orientation.

Once synthesized in the liver, the primary role of CRP is modulating the inflammatory or immune response in the body [55]. CRPs are a member of non-antibody proteins that play a significant role in the development of the body’s initial immune response. The initial stages of CRP activation of the immune response requires binding from several ligands, such as damaged tissues, bacteria, and fungi, which can be categorized by whether the ligands have a similar structure to CRP and contain polycationic properties or carbohydrates with D-galactose-related structures [349,350,351].

CRP activation increases several immune responses including preventing adhesion of neutrophils to endothelial cells, initiating the classical complement pathway through C1q, increasing opsonophagocytosis, and increasing vascular wall inflammation through upregulating E-selectin or ICAM-1 proteins. Specifically, CRP functions as an innate opsonin and promotes complement activity towards pathogens by binding it to phosphatidylcholine. CRP also binds the Fcγ receptor, which serves as a bridge between inflammation, innate immunity, and complement activity [55]. Furthermore, CRP’s ability to inhibit endothelial NO production and activation of monocytes in metabolic syndrome further suggests a causal role in inflammation. There is now mounting evidence that it also plays crucial roles in inflammatory processes and host defenses against infection [335,352,353].

In addition to modulating the immune response, CRP contributes to atherogenesis by activating the complement system and facilitating the uptake of low-density lipoprotein (LDL) in macrophages [354]. Low-density lipoprotein retention and modification in arterial walls is believed to be at the root cause of atherosclerosis. To create foam cells, modified LDL is swallowed by macrophages, which aids in the formation of atherosclerotic lesions [355]. Modified LDL is a significant CRP–ligand with biological importance. CRP rapidly binds to modified (e.g., oxidized and enzyme-treated) LDL, but not to natural LDL in a Ca^2+^-dependent manner [356,357,358]. The PCh-binding site in CRP interacts with the PCh and cholesterol moieties on LDL to mediate binding of CRP to LDL. CRP is thus capable of covering certain properties of modified LDL, such as complement activation, which protects the host from complement activation by LDL. The known ligand-binding specificities of CRP serve as justification for its accumulation in atherosclerotic lesions. At normal pH, CRP binds to molecules and cells with exposed phosphocholine moiety, such as apoptotic cells and necrotic cells found in cardiac infarcts, in a calcium-dependent manner [346,358]. However, several studies have come to different conclusions on whether CRP is involved in the pathogenesis of atherosclerotic lesions. In addition to CRP’s multiple functions in the cell, it is possible that differences in solutions that are free of calcium or methods that remove calcium during an experiment may influence the activity and the species of CRP (nCRP or mCRP) present. This difference could explain the conflicting experimental results in the contribution of CRP to atherosclerosis and, ultimately, CVD.

Despite not having a specific diagnostic function, CRP is very helpful in clinical practice since variations in its serum or plasma level reflect the presence and potency of a variety of immune processes. Therefore, estimating CRP activity may aid in distinguishing between infectious and noninfectious illnesses, assessing prognosis, predicting future risk, and evaluating therapy response. Additionally, few immunosuppressive or anti-inflammatory medications directly affect CRP levels unless they have an impact on the course of an underlying illness. As a result, CRP is now frequently utilized in clinical practice and has recently been recognized as a more significant marker of acute-phase reactions. Circulating CRP levels exhibit strong correlations with other inflammatory markers, some of which exhibit analogous, but typically less substantial, predictive relationships with coronary events. The fact that CRP is a particularly stable analyte in serum or plasma makes it a reliable, highly standardized, reproducible, and easily accessible analyte in clinical practice. None of the other systemic markers of inflammation, whether upstream cytokine mediators, other sensitive acute-phase proteins, or other multifactorial measures, have similar sensitivities or specificities to CRP. Therefore, CRP’s inherent biological characteristics as an acute-phase reactant are particularly advantageous for its application as a sensitive quantitative measurement of inflammatory processes within the body [359,360].

In clinical practice, CRP levels are measured by the highly sensitive assay (hsCRP), which is one of the most extensively validated. The hsCRP assays can be precisely quantified from fresh or frozen plasma and have been standardized across various commercial platforms [361,362]. In the search for the best biomarker for predicting the risk of CVD globally, the hsCRP is the biomarker that has been investigated the most. In combination with a parent’s history of an early myocardial infarction, it has been incorporated into the Reynolds Risk Scoring system for women’s global CVD risk prediction, and it can more accurately reclassify 50% of all women into the ATP III intermediate risk category (annual CVD risk of 5–10%) and 10–20% into higher or lower 10-year risk categories. In addition, a relative risk of coronary event is two times higher for patients with a CRP concentration >2.4 mg/L. However, the increase in CRP cannot identify a specific cardiovascular etiology for the increased CVD risk, which could be related to several processes, such as inflammation in atherosclerotic plaques, total atherosclerotic burden, other inflammatory or metabolic processes, or metabolic or patient-specific higher CRP responsiveness to prevalent endogenous or environmental acute-phase stimuli [363]. Therefore, CVD is not exclusively linked to CRP; other inflammatory systemic indicators exhibit comparable correlations, albeit weaker ones than those of CRP.

It is likely that hsCRP can improve CVD-R prediction over the clinical diagnostic criteria as well as serum lipid measurements, perhaps because it represents an integrated measure of multiple types of stress [364]. However, not all studies have confirmed improved prediction of CVD-R in metabolic syndrome by CRP measurements and the predictive value beyond conventional criteria may be small [55,364,365,366,367,368,369,370]. Inclusion of CRP increases the predictive ability of the Framingham Research Study (FRS) in intermediate-risk populations and adds significantly to the predictive power of TC/HDL-C [371]. In addition, CRP has been suggested to be useful in predicting the CV risk of individuals without known CV risk [372,373]. In a systematic review of prospective studies that included 84,063 individuals, with 11,252 coronary events, CRP increased the predictive value of FRS by 3–8% only [372]. While the biomarker C-reactive protein is considered one of the best predictors of CVD-R, it is significantly lower in men than women [371,374]; if CRP indeed plays a direct causative role in CVD, as previously suggested [55,371], the opposite would be expected given that CVD-R is greater in men than women. Certainly, significantly elevated CRP above the normally higher age-adjusted baseline in women does increase their CVD-R. The lack of correlation of baseline CRP level (absolute rather than relative) with CVD thus implies that increased serum levels of CRP are a consequence rather than cause of CVD in both genders. Serum CRP remains the most widely utilized routine test for directly measuring OX-S or inflammation for determination of an accurate individual risk [371,375]. CRP is well correlated with overall population risk for CVD-R but is clearly not sufficient to measure accurately an individual risk, unless linked with serum lipoprotein measurements. Indeed, some individuals with high HDL-C and CRP have a 2.4-fold higher CVD-R if the CETP gene is mutated [376,377].

Given the correlation of CRP levels with CVD, large randomized clinical trials, most notably the JUPITER trial, investigated whether reductions in CRP using statins may reduce CVD-R. Previous studies have shown that statins are also beneficial for people with normal LDL-C levels who simultaneously have elevated oxidative stress or inflammation, as indicated by raised CRP values. The multinational placebo-controlled JUPITER trial’s findings, which looked at how rosuvastatin affected deaths, MIs, or strokes in 17,802 men and women without a history of heart disease and who had normal LDL (130 mg/dL) and abnormal CRP (>2 mg/L), are the strongest support for this association [55,364,378,379]. The JUPITER cohort had a baseline 36% higher risk of CVD than age-matched controls without increased CRP, and hence it is uncertain whether the results or cost-effectiveness of the treatment are applicable to truly low-risk populations [12,380,381,382,383]. Rosuvastatin provided benefit in those with elevated CRP in both groups [379]. A meta-analysis of 54 prospective cohort trials shows that increase in CRP confers a 1.3–1.5-fold risk, whereas the same increase in cholesterol confers a 1.1–1.3-fold risk [379]. Despite its limitations, the JUPITER study provides a clear rationale for the use of statins in asymptomatic persons with intermediate CVD-R (10–20% per 5 years) who have normal LDL-C but evidence of increased inflammatory and oxidative stress as reflected by an elevated CRP level [12,379]. In addition, CRP levels increases the stroke risk by 2-fold, which is independent of smoking BP, HDL-C, and DM [12,383].

Summary: CRP is the most extensively validated inflammatory biomarker for CVD-R prediction, with hsCRP incorporated into the Reynolds Risk Score and shown to confer a 1.3–1.5-fold CVD risk per unit increase. The JUPITER trial demonstrated that rosuvastatin reduced CVD events in individuals with normal LDL but elevated CRP (>2 mg/L), providing rationale for statin use in intermediate-risk asymptomatic persons with evidence of increased inflammation. However, CRP lacks cardiac specificity, baseline levels are paradoxically higher in women despite their lower CVD-R, and its independent predictive value beyond conventional risk factors remains modest (3–8% improvement over FRS), suggesting it reflects a consequence rather than a direct cause of cardiovascular disease.

## 11. IL-6

Small (15–20 kilodalton) soluble proteins called cytokines send signals to distant organs or transduce signals in nearby cells. Most cytokines interact with receptors and then send intercellular signals to their target cells to modulate several functions, including immunity, development, metabolism, aging, and cancer progression. Among these cytokines, Interleukin 6 (IL-6) signaling can exert both pro- and anti-inflammatory effects through interactions with its receptors, thus controlling the balance between pro- and anti-inflammatory cytokines in a highly context-dependent manner. IL-6, also known as B cell stimulatory factor 2, was first discovered by Kishimoto’s group in the 1970s as a soluble protein produced by T cells that stimulates the differentiation of B cells into antibody-producing cells [384]. Later, it was discovered that hepatocyte-stimulating factor and plasmacytoma growth factor were shown to be IL-6 [385]. After IL-6′s receptor and signaling molecules were cloned, they were each cloned one at a time. In 1988, the human IL-6R was first cloned [386]. A 130-kD glycoprotein (gp130, commonly known as CD130) was shown to be a further receptor component that serves as an IL-6 signal transducer in 1990 by Kishimoto’s team [387].

IL-6 serves as a mediator for alerting cells to some external stressor. Pathogen-recognition receptors (PRRs) of immune cells such as monocytes and macrophages detect the foreign pathogen’s signature, known as pathogen-associated molecular patterns, in the infected lesion [388]. These PRRs include Toll-like receptors (TLRs), retinoic acid-inducible gene-1-like receptors, nucleotide-binding oligomerization domain-like receptors, and DNA receptors. These signaling pathways increase the transcription of the mRNA for inflammatory cytokines like IL-6, tumor necrosis factor alpha, IL-1beta, and NF-kB. In cases of tissue damage, IL-6 also sends out a signal. In noninfectious inflammations like burns or trauma, damage-associated molecular patterns (DAMPs), which are generated from damaged or dying cells, directly or indirectly increase inflammation. A rise in serum IL-6 levels occurs prior to an increase in body temperature and serum acute-phase protein concentration during sterile surgical procedures [389]. Mesenchymal cells, endothelial cells, fibroblasts, and numerous other cells, in addition to immune-mediated cells, participate in the production of IL-6 in response to various stimuli. Subsequent studies demonstrated that several transcription factors control the transcription of the IL-6 gene.

Since the crystal structures of IL-6, IL-6R, and the IL-6 signaling complex were all solved in 2003 [390,391,392,393,394], IL-6 is the member of this family that has been structurally most thoroughly characterized. With the anticipated up–up–down–down arrangement of alpha-helices and an extra short helix in the CD loop, IL-6 is a typical four-helical bundle cytokine. An N-terminal Ig-like domain and two Fn3 domains make up the three domains that make up the extracellular region of the IL-6R. IL-6 sends its signals via a cell-surface type I receptor complex made up of the gp130 signaling subunit and the ligand-binding glycoprotein known as the IL-6 receptor (IL-6R or CD126). The receptor consists of three domains in it: D1, D2, and D3. It consists of an Ig-like domain, a membrane-spanning region, a tryptophan-serine-X-tryptophan-serine (WSXWS) motif in the cytokine receptor family domain, which is predicted to serve as a ligand interaction site [395], and a short cytoplasmic domain that is not necessary for signal transduction. The D1 domain, sometimes referred to as the Ig domain, is connected to the CBD (cytokine-binding domain), which is made up of the D2 and D3 domains. There are two types of IL-6R: membrane-bound (mIL-6R) and soluble (sIL-6R). Once IL-6 binds to a receptor, it can cause several functions within the cell.

Specifically, IL-6 can stimulate the manufacture of acute-phase proteins in hepatocytes, including CRP, serum amyloid A, fibrinogen, and hepcidin, while inhibiting albumin formation [396]. The growth of effector T cells and the generation of antibodies are both stimulated by IL-6, which also has a significant impact on acquired immunological response. Additionally, IL-6 can encourage the proliferation or differentiation of a variety of nonimmune cells. Through the modulation of their transporters, IL-6 is also implicated in the regulation of serum iron and zinc levels. Regarding serum iron, IL-6 stimulates the development of hepcidin, which prevents ferroportin 1 from transporting iron through the stomach, lowering serum iron levels. This indicates that hypoferremia and anemia related to chronic inflammation are caused by the IL-6–hepcidin axis. Additionally, IL-6 produces the hypozincemia seen in inflammation by increasing the expression of the zinc importer ZIP14 on hepatocytes. When IL-6 enters the bone marrow, it encourages the maturation of megakaryocytes, which results in the production of platelets. The dysregulated continuous production of IL-6 causes the onset or development of numerous diseases, such as CVD, due to its pleiotropic action.

IL-6 concentrations are normally low in healthy patients who do not have inflammation, ranging between 0.2 and 7.8 pg/mL [397,398,399,400]. However, it can reach as high as 1600 pg/mL in sepsis. Age, diabetes, and acute exercise all alter IL-6 concentrations. It is crucial to be able to properly quantify IL-6 over a wide dynamic concentration range and to have faith in the face validity of the measure, which means that it is measuring what it is supposed to measure, to understand the various biological pathways in which IL-6 is involved. However, one of the main costs for measuring IL-6 is the higher cost compared to other biomarkers [401]. IL-6 also has a short half-life and is difficult to accurately assay. This makes it very challenging to determine an individual’s IL-6 level and correlate it to their CVD [401]. The main benefit for IL-6 serum measurements is its assessment of treatment efficacy [402,403]. There is a dose-dependent relationship between IL-6 and IL-6 receptor levels in CVD, whereby increased levels of soluble (i.e., circulating) IL-6R leads to decreased levels of membrane-bound IL-6R, which in turn lowered signaling and subsequent inflammation (e.g., reduced CRP levels) [402,403]. As such, IL-6 can be used to assess CVD-R, particularly in high-risk patients.

IL-6 levels in serum correlate directly with CVD [404,405,406,407,408,409,410,411,412,413,414,415,416,417,418,419]. Higher levels of IL-6 correlate with poor outcomes in hospitalizations with unstable angina [420,421] and poor LVF [422]. Cardiac morbidity and mortality are higher in those with high IL-6 after myocardial infarction [410,416,418,419,423,424,425]. IL-6 is an independent predictor of myocardial infarction mortality. In addition, CVD patients with high IL-6 levels are associated with a higher rate of restenosis [414,426]. Given the association of IL-6 and CVD, several clinical trials have examined whether targeting IL-6 would improve clinical outcomes for patients with CVD, particularly myocardial infarction [427,428,429]. Comatose patients who had been revived from an out-of-hospital cardiac arrest experienced a significant decrease in systemic inflammation and myocardial damage after receiving tocilizumab (monoclonal IL-6 inhibitor) treatment [428,429]. In addition, tocilizumab was effective at reducing CRP levels in whole blood, which was significantly attenuated by IL-6R-inhibition in NSTEMI patients [427]. Together, our findings offer proof that IL-6 and IL-6R play a direct causative role in the onset of CVD and point to a potential therapeutic target for CVD prevention.

Summary: IL-6 is a pleiotropic cytokine that drives acute-phase protein synthesis (CRP, fibrinogen, hepcidin) in hepatocytes and independently predicts myocardial infarction mortality and CVD outcomes. Clinical trials with tocilizumab (IL-6 receptor inhibitor) have demonstrated reductions in systemic inflammation and myocardial damage, supporting a direct causative role for IL-6 signaling in CVD. However, its clinical utility as a routine biomarker is limited by its short half-life, assay difficulty, and higher measurement cost compared to other biomarkers.

## 12. TNFa

The original idea of an anti-tumoral response in the immune system was first proposed by the physician William B. Coley [430,431,432]. William Coley, a doctor in New York, noticed that several cancer patients went into remission following bacterial infections in the early 1900s. Dr. Coley began simulating bacterial diseases by purposefully giving patients different combinations of germs in a series of daring experiments. Prior to the development of chemotherapy and radiotherapy, one of these mixes, combining Streptococcus pyogenes and Serratia marcescens, was widely utilized in the United States (US) after it was shown to have therapeutic activity. The name “tumor necrotizing factor” was first used in 1962 for tumor (sarcoma 37) regression activity induced in the serum of mice treated with *Serratia marcescens* polysaccharide. Further studies examining this unknown factor were further elucidated by Carswell et al. in 1975 [432]. It was in this year that the unknown factor, known previously as “Coley’s toxins”, was given the name tumor necrosis alpha (TNFa) [431,432].

In the 1980s, Aggarwal et al. identified and cloned two different TNFa using human lymphoblastoid cell line RPMI 1788 [433]. Specifically, it was shown that the TNFa was composed of 233 amino acids, with the first 76 amino acids being its leader sequence [434,435,436]. It was interesting to discover that the same sequence belonged to cachectin, another protein linked to cancer. The effects of cachectin on peripheral tissues and the central nervous system have been shown to mediate weight loss and change normal metabolic priorities. Subsequent studies found that TNFa was reported to cause hypotension, metabolic acidosis, hemoconcentration, and mortality from respiratory arrest within minutes to hours when given in doses that were close to the endogenous levels that were seen in response to endotoxin [437,438,439,440]. This pattern mimicked the symptoms of sepsis. Following infusion, hyperglycemia and hyperkalemia were also noted. On visual and histopathologic inspection during necropsy, extensive pulmonary inflammation and bleeding were visible, coupled with ischemic and hemorrhagic lesions of the gastrointestinal tract and acute renal tubular necrosis. TNFa thus proved to be a powerful tumor regression mediator that induces cachexia and contributes to septic shock.

Ligands, such as TNFa, are expressed in soluble and transmembrane forms. Although soluble ligand is associated with TNFa’s pathogenic effects, the transmembrane form of the ligand seems to mediate therapeutic effects. Ligands, such as TNFa, are only produced as soluble proteins because they lack the transmembrane domain. Tumor cell-produced TNFa mainly exerts its autocrine and paracrine effects through TNFR1. There are instances of TNFa-mediated reverse signaling when it interacts with its receptor. Several studies have been done on the process by which TNFa and the other members of its family transduce signals from target cells [441,442]. The fact that the 29 different receptors are interacted with by the 19 members of the TNF superfamily suggests that at least some of the ligands must interact with multiple receptors. TNF receptor 1 (TNFR1) and TNFR2, for example, are two different receptors that TNF is known to interact with. It has been demonstrated that BAFF binds to three different receptors on B cells, including the transmembrane activator and CAML interactor (TACI), the B-cell maturation protein (BCMA), and the BAFF receptor (BAFFR). Death receptor 4 (DR4), death receptor 5, decoy receptor 1 (DcR1), decoy receptor 2, and osteoprotegerin are some of the receptors that TRAIL has been identified to bind to (OPG).

Both a soluble and membrane-bound version of TNF-alpha can be discovered. TNFa-converting enzyme (TACE), a metalloproteinase that is a member of the ADAMs family of disintegrins, cleaves TNFa from its membrane forms to produce the soluble plasma form [433,443,444,445,446,447,448]. To activate receptors, soluble TNF-alpha, a 17 kDa protein of 157 amino acids, forms a homotrimer. Natural killer (NK) cells, T lymphocytes, and activated macrophages are the major producers of TNFa. TNF-beta, formerly known as Lymphotoxin, is a similar but different cytokine that has been shown to share some of the activities of TNFa. The TNF family now consists of 19 members along with 29 receptors that have been identified. One of two groups—those with an intracellular death domain (DD) and those without—comprises most of the TNF superfamily receptors. The DD, a section of about 45 amino acids, is necessary for the recruitment of other proteins that cause cell death.

TNFa is the most polytropic cytokine and is involved in both anti-inflammatory and tissue healing processes [449,450,451]. Tumorigenesis is aided by its actions at low expression levels. TNFa, IL-1, and other chemokines are primarily produced by activated macrophages and other myeloid lineage cells in response to inflammatory stimuli, attracting and activating neutrophils and monocytes to the tumor site. TNFa also causes inflammation, stimulates the vascular endothelium, directs the recruitment of immune cells into the tissue, and encourages tissue deterioration. Through the formation of reactive oxygen intermediates and signaling through ceramide, the membrane version of TNFa on tumor cells interacts primarily with TNF-R2 to trigger clearance of pro-tumor suppressor cells. Several studies indicate that TNFa is possibly cardioprotective for the failing myocardial while being cardiotoxic for the healthy myocardium [452,453,454]. The main cause of cardiotoxicity is TNFa-induced cardiomyocyte apoptosis, whereas the main cause of cardioprotection is TNFa-induced ectopic production of keratin 8 and keratin 18 in cardiomyocytes. By altering lipid metabolism, activating endothelial cells, and causing vascular inflammation, TNFa is likely implicated in the etiology of atherosclerosis in addition to its involvement in cardiac diseases. Given the importance of TNFa in cardiovascular disease, there has been a push to develop a method for measuring serum levels of TNFa [455].

In particular, a quick and straightforward test method is required to screen many substances for novel inhibitors that prevent TNFa secretion [456]. The most popular TNFa-measurement techniques now available are the radioisotope-labeled immunological assay and ELISA (Enzyme-Linked ImmunoSorbent Assay). The lengthy incubations and several wash stages in these assays, along with the production of a significant amount of radioactive waste, limit the throughput of the screen. For this investigation, we used the HTRF-based and AlphaLISA-based assay formats to produce two homogeneous TNFa assays. Both assays have been validated on a quantitative high-throughput screening (qHTS) platform and downsized into a 1536-well plate format. As a result, TNFa has not been routinely adopted in clinical practice for assessing cardiovascular risk.

Several clinical studies have linked TNFa to an increased CVD risk, particularly among patients with a history of diabetes, obesity, atrial fibrillation, psoriatic arthritis, rheumatoid arthritis, and arthritis [457,458,459,460,461,462,463,464,465,466,467,468,469,470]. Among CVD patients, elevated levels of circulating TNFR1 and TNFR2 were linked to higher risks of cardiovascular events and mortality. However, it seems that there is minimal value in TNFR1 and TNFR2 measurement to enhance risk prediction in these patients [471]. Further clinical studies and technological advancements are required before TNFa can be applied in high-risk CVD patients.

Summary: TNFα is a highly pleiotropic cytokine with dual cardioprotective and cardiotoxic properties, inducing cardiomyocyte apoptosis in healthy myocardium while promoting protective keratin expression in failing myocardium. Elevated circulating TNFR1 and TNFR2 levels are linked to higher cardiovascular events and mortality in patients with diabetes, obesity, atrial fibrillation, and inflammatory arthritides. However, TNFα has not been routinely adopted for clinical CVD risk assessment due to measurement challenges and minimal incremental value for risk prediction.

## 13. Lipokines

### 13.1. Leptin

In a meta-analysis of over 21,000 patients with over 2000 CVD events, elevated leptin levels conferred a 1.36- and 1.5-fold increased odds ratio for CVDR in men and women, but the results were marginally significant. Interestingly, adjustment for socioeconomic status yielded a statistically significantly higher risk of 1.47 for men [472]. A small cross-sectional study found that obese people have high serum levels of leptin [473]. Though recombinant leptin causes weight loss in mice, it is ineffective in humans because of the presence of endogenously high levels of structurally normal leptin, essentially leptin resistance [474]. Leptin levels are increased and directly correlated with BMI in obese humans, though there is considerable inter-individual variation. Weight loss does reduce leptin levels [475]. A study comparing lean and 50 obese subjects showed significant age-adjusted increases in insulin resistance and Adipocyte Fatty Acid Binding Protein (AFABP). Though adiponectin, leptin, and resistin levels were lower, and CRP, retinol-binding protein 4 (*RBP4*), and fibroblast growth factor 21 (FGF21) were higher in the obese, the difference was not significant upon age adjustment. Chemerin and FGF-21 levels were not affected in obese subjects. However, this study was not well balanced for gender, with females predominate in the lean and males in the obese population [476]. Plasma levels of adiponectin, adipsin, resistin, leptin, or plasminogen activator inhibitor-1 (PAI-1) were not correlated with CVD-R in a large European study. In contrast, CRP and IL-6 were independent CVD-R factors [477]. Serum leptin concentrations are directly correlated with BMI, sedentary behavior, circulating insulin, C-peptide, and soluble tumor necrosis factor receptor-1 (sTNFR1) and soluble tumor necrosis factor receptor-2 (sTNFR2) levels. After adjustment for BMI, it correlates independently only with HgbA1C and sTNFR1, but this correlation is applicable only in those with BMI > 25 [33]. Due to its cost and few prospective studies on leptin levels and CVD, leptin is currently not a routine lab used for assessing CVD risk. Additional prospective research is required to determine whether lower leptin levels and higher insulin sensitivity can enhance primary endpoints like the prevalence of diabetes mellitus and the results of cardiovascular events [478].

### 13.2. Adiponectin

Visceral adiposity is linked to low adiponectin levels in obese and diabetic individuals [479,480]. Adiponectin levels are inversely and independently associated with a lower risk for developing DM [481]. However, high adiponectin levels in diabetics and individuals with chronic inflammatory diseases render adiponectin a poor biomarker and raise questions regarding its anti-inflammatory role [482]. Higher risk of DM in those with low adiponectin appears to be relevant only in those with existing insulin resistance. Thus, an independent role of adiponectin in mediating DM or insulin resistance is unlikely [483]. Therefore, low adiponectin levels are a consequence rather than a cause of insulin resistance [484]. Additionally, the association of low adiponectin with the risk of DM appears to depend on concomitantly high leptin [485]. The association of adiponectin with insulin resistance has not been uniformly confirmed [486]. Adiponectin is currently not used routinely in clinical assessment of CVD. Instead, adiponectin has been used to assess the cardioprotective effects of medications, such as thiazolidinedione [487]. Further studies will be needed to assess the long-term prognostic efficacy of adiponectin [487].

### 13.3. RBP4

Serum RBP4 levels are higher in men, increased in T2DM, and correlate with BMI and multiple measures of insulin resistance [488,489,490]. RBP4 expression in adipose tissue and single nucleotide polymorphisms (SNPs) in RBP4 are associated with risk for T2DM and a greater visceral adipose tissue/subcutaneous adipose tissue (VAT/SAT) ratio [491,492,493]. Its levels are correlated with markers of systemic inflammation and reduced upon weight loss due to bariatric surgery [491,494]. Several studies have not confirmed the relationship between serum levels of RBP4 and obesity or insulin resistance [493,495,496,497], but this may be due to methodology or varying levels of renal dysfunction that can affect RBP4 levels [498,499]. In one study, RBP4 was higher in the obese, but the difference was not significant upon age adjustment [476]. RBP4 levels correlate with many serum inflammatory markers, and serum levels are increased in obesity [490], but do not correlate well with T2DM [500]. RBP4 is a new biomarker for CVD in need of additional prospective studies to determine its accuracy as a marker for CVD [501]. Additional research is needed to confirm whether RBP4 is a risk factor and a diagnostic marker for CVD in high risk patients [501].

### 13.4. Resistin

A small cross-sectional study found that obese people have high serum levels of resistin [473]. Obese humans have high resistin levels in tissue and blood. It correlates with the severity of coronary atherosclerosis and CHF. Resistin levels correlate with obesity and PPARg levels [502]. In human studies, there is no correlation of serum resistin levels with DM, obesity, or insulin resistance [503]. However, resistin levels do not correlate with insulin resistance [504]. Resistin appears to be correlated with the presence of atherosclerosis [505]. Resistin correlates with the presence of CAD and is a predictor of coronary restenosis after MI in DM [506]. In humans, resistin correlates with obesity as well as BMI [507,508]. SNPs in nucleobindin-2 (NUCB2) are correlated with adult [509] and childhood [510] obesity and decreased levels in diabetics may be responsible for their hyperphagia [511,512]. Resistin levels correlate with CRP, IL6, and TNFa [513]. In a study of 2131 FSH offspring without T2DM, resistin was not correlated independently with insulin resistance [486]. Similar to RBP4, resistin is a new biomarker for CVD that requires additional prospective studies to validate its use for CVD [514,515]. In addition, the mechanistic processes by which resistin can influence several organs and tissues is still being investigated [514,515].

### 13.5. Monocyte Chemoattractant Protein-1 (MCP-1)

Serum levels of MCP1 are correlated with multiple risk factors for MI and atherogenesis, including age, cigarette smoking, TG, BMI, and waist circumference. The MCP1-2578G allele affects the binding region for C-C chemokine receptor-like 2 (CCRL2) and results in increased MCP1 signaling [516]. Serum MCP1 levels are not uniformly increased in obesity; the association appears to be dependent on ethnicity, gender, and menopausal status [517]. MCP1 is a new biomarker for CVD that requires additional prospective studies to validate its use for CVD. However, a recent study in 2021 showed that, in community-dwelling adults without overt cardiovascular illness, higher circulating MCP-1 levels are linked to higher long-term cardiovascular mortality [518]. These results add to the evidence that MCP-1 signaling has a significant role in cardiovascular disease [518].

### 13.6. P-Selectin

P-selectin was associated with metabolic syndrome risk (weakly, 1.06-fold) independent of other FRS risk factors, but CRP, CD40 ligand, ICAM1, IL-6, MCP1, OPG, TNFa, and TNF beta were not. Insulin resistance is correlated with CRP, particularly in women [365]. P-selectin is a new biomarker for CVD that requires additional prospective studies to validate its use for CVD.

### 13.7. Lipocalin

Lipocalin serum levels are elevated in db/db mice, and rosiglitazone normalizes these levels. In a human study of 229 subjects, lipocalin levels correlated with CRP independent of age, gender, or weight and correlated with insulin sensitivity [519]. Lipocalin serum concentrations are correlated with obesity, triglycerides, and insulin resistance and are inversely correlated with HDLC [519,520]. Lipocalin is a new biomarker for CVD that requires additional prospective studies to validate its use for CVD. However, a recent study examining lipocalin showed that serum levels may be useful for risk stratification in advanced and early peripheral artery disease, which is a known risk factor for CVD. Therefore, additional studies on lipcalin and CVD may provide additional data to support its use as a prognostic biomarker for moderate- to high-risk CVD patients [521].

### 13.8. TGF Beta

TGF-beta levels do not correlate with CRP, IL-6, IL-18, or MCP-1, but high levels are associated with a marginally significantly higher risk of DM [522]. Though cell and animal evidence is convincing [523], associations between serum levels and obesity, risk of DM, or cardiovascular events is not convincing for a role as an anti-inflammatory cytokine clinically [524]; it may simply be a marker of inflammation. TGF-beta is a new biomarker for CVD that requires additional prospective studies to validate its use for CVD.

### 13.9. Chemerin

Serum chemerin levels are poor predictors of CVD-R [525]. Chemerin levels are increased in obesity [526] and early metabolic syndrome [527]; however, chemerin was not affected in obese subjects [476]. Weight loss reduces serum chemerin levels [528]. Chemerin is a new biomarker for CVD that requires additional prospective studies to validate its use for CVD. A recent prospective cohort study demonstrated a significant, independent relationship between chemerin and CVD [529]. A large positive correlation was also discovered for T2DM; however, a sizable percentage of it could be attributed to body mass index and waist circumference [529]. Therefore, additional studies are needed to assess whether development of CVD may be directly linked to chemerin and, if so, whether it can be used as a reliable biomarker for CVD [529].

### 13.10. Other Cytokines and Lipokines

Serum secreted-frizzled related protein 5 (SFRP5) levels have been positively and negatively correlated with obesity [530,531]. However, the very low expression of SFRP5 human fat makes it unlikely to plays a causative role in human obesity or insulin resistance [532,533]. Low levels of omentin in diabetics correlate with a higher rate of post-MI complications in diabetics [534]. Omentin levels are closely correlated with adiponectin, being lower in obesity and insulin resistance [535]. Visfatin correlates with IL-6, CRP, and MCP1, but correlations with atherosclerosis, endothelial dysfunction, or DM are inconsistent [536]. BMI and insulin resistance are proportional to visfatin expression in VAT and inversely proportional to that in SAT; total visfatin does not correlate with BMI or insulin resistance [537]. Apelin levels are increased in diabetics, but the correlation is weak [538]. Serum levels of vaspin correlate directly with BMI, and high levels are associated with T2DM and insulin resistance [539,540,541]. The correlation of neurofibromatosis 1 (NF1) with obesity is in a very narrow range of weight, and only in men [542,543]. DPP4 release from adipose tissues correlates with markers of metabolic syndrome [544]. Serum Interleukin 1 receptor antagonist (IL1RA) is an independent factor that relates obesity to insulin resistance [545].

Summary: Among the lipokines, leptin shows a modest 1.36–1.5-fold CVD-R association, while adiponectin, resistin, RBP4, MCP-1, chemerin, lipocalin, and others demonstrate variable and often inconsistent correlations with CVD-R, obesity, and insulin resistance across studies. Most lipokines remain investigational biomarkers requiring additional prospective validation, with MCP-1 showing the most recent promise through its independent association with long-term cardiovascular mortality in community-dwelling adults.

## 14. Cell Adhesion Molecules

Endothelial cells are the primary source of ICAM and VCAM [513]. Resistance levels are correlated with VCAM and ICAM expression [546]. Mortality from MI is not predicted by serum-soluble intercellular adhesion molecule (sICAM), soluble vascular cell adhesion molecule-1 (sVCAM-1), or P-selectin levels (*n* = 263) [547]. Mortality from MI is not predicted by sICAM, sVCAM, or P-selectin levels (*n* = 263) [547]. According to recent studies, sVCAM-1 may be a biomarker for AF, POAF, and cardiovascular death in patients undergoing coronary artery bypass surgery who had coronary artery disease [548]. Before creating a recognized biomarker, however, various aspects of sVCAM-1, like the specificity, cut-off levels for each type of disease, and measurement standards, need to be addressed [548]. In conclusion, sVCAM-1 can be thought of as a biomarker in development, and further study is required to clearly define its function as a biomarker for AF.

Summary: Soluble ICAM, VCAM-1, and P-selectin levels do not predict MI mortality. sVCAM-1 shows emerging potential as a biomarker for atrial fibrillation and cardiovascular death in coronary artery bypass patients, but specificity, cut-off values, and measurement standards require further definition before clinical adoption.

## 15. Oxidized Lipids

Since oxidation of eicosanoid fatty acids is a common chemical denominator of all oxidative stress, measurements of lipid-hydroperoxides and their metabolites could function as improved biomarkers. Several lines of evidence suggest the benefit of oxidative stress biomarkers in improving CVD-R prediction beyond the FRS criteria [19]. Elevated levels of several eicosanoid metabolites, 9-Hydroxyeicosatetraenoic acid (9-HETE) and F2-isoprostanes are strongly associated with angiographic evidence for coronary artery disease, but their inclusion in multivariate risk models does not improve the ROC of the traditional FHS study risk factors. F2-isoprostanes (i.e., 8-epi-PGF2a) are established biomarkers of lipid peroxidation.

Prostaglandin F2 Alpha (PGF2a) levels are increased in multiple insulin resistance-associated states and rise steadily with age. Its levels are generally closely correlated with inflammatory markers, including CRP, IL-6, and TNFa. Weight gain is associated with increases and weight loss with decreases in all four markers. Thus, PGF2a is an integrated marker of oxidative stress, inflammation, BMI, and age. Neither antioxidants nor anti-inflammatory agents have a significant impact on the prevention or treatment of obesity. Thus, the cause–effect relationship between obesity and oxidative stress is far from clear [35].

The serum thiols, including homocysteine, glutathione, and cysteinyl glycine, are also correlated with CVD-R but have not been shown to improve risk prediction beyond the FRS. Recent evidence indicates that the activity in serum of the enzyme paraoxonase-1 (paraoxonase arylesterase, PON1) correlates with CVD-R [549]. Several pre-clinical observations suggest a potential mechanism for the function of PON1 as an antioxidant enzyme. PON1 catalyzes the hydrolysis of oxidized phospholipids. It also hydrolyzes homocysteine thiolactone, a pro-atherogenic metabolite of homocysteine [550]. The ability of PON1 to protect LDL from oxidation is not established since oxidized lipids inhibit its arylesterase activity [551]. PON1 expression is inhibited in vitro by IL-1, IL-6, TNFα, and oxidized phospholipids [552,553,554,555]. CVD-R, obesity, low HDL-C, renal disease, T2DM, smoking, and hepatic cirrhosis have been correlated with low PON activity [556,557,558,559,560,561,562,563,564,565]. Simvastatin increases PON1 promoter activity in a dose-dependent manner, upregulating the binding of nuclear transcription factors Sp1 and sterol regulatory element-binding protein-2 (SREBP-2) to the promoter [566].

Summary: Lipid peroxidation biomarkers such as F2-isoprostanes and 9-HETE correlate with angiographic coronary artery disease, and PGF2α serves as an integrated marker of oxidative stress, inflammation, BMI, and age, though none significantly improve CVD-R prediction beyond the Framingham Risk Score. Paraoxonase-1 (PON1) shows promise as an antioxidant enzyme whose activity correlates with CVD-R and is upregulated by statins, but its protective role against LDL oxidation remains unestablished.

## 16. Free Fatty Acids

Free fatty acids (FFAs) play a key role in promoting obesity, inflammation, and oxidative stress. They activate TNFa, MCP1, and IL-6 secretion from subcutaneous and omental fat. FFAs inhibit the insulin-mediated shutoff of gluconeogenesis [567]. IGF1 inhibits FFA-induced TNFa, secretion from subcutaneous fat only. FFA activates Jun N-Terminal Kinase (JNK) in subcutaneous as well as omental fat. However, IGF1 inhibits JNK activation in subcutaneous fat, while activating it in omental fat [568]. High fat intake can overwhelm fatty acid re-esterification in cells and contribute to elevated serum FFA levels seen in obesity. High levels of FFAs can acutely induce insulin resistance. Diacylglycerol transferase catalyzes the formation of triglycerides from diacylglycerol (DAG); its over-expression reduced FFA accumulation, and deficiency causes an increase in FFA levels. Reduced esterification of FFA to TG increases cellular DAG levels resulting in activation of protein kinase c beta 2 (PKCb2) and protein kinase C zeta (PKCζ). The consequent activation of nuclear factor kappa-light-chain-enhancer of activated B cells (IkB and NFkB) results in the expression and secretion of TNFa, IL-6, IL-1b, and MCP1, all of which can activate JNK and SOCS, resulting in insulin resistance through inhibition of insulin receptor binding to insulin receptor substrate 1 (IRS1). JNK is also activated by reactive oxygen species (ROS) that result from oxidative endoplasmic reticulum stress caused by the inhibition of NADPH oxidase by FFA.

Toll-like receptor 4 (TLR4) contributes to activating this resistance pathway by promoting PKCb2-mediated IkB activation [46]. Free fatty acids (FFAs) activate TNFa, MCP1, and IL-6 secretion from subcutaneous and omental fat. IGF1 inhibits FFA-induced TNFa secretion from subcutaneous fat only. FFA activates JNK in subcutaneous as well as omental fat. However, IGF1 inhibits JNK activation in subcutaneous fat while activating it in omental fat [568]. FADS1 and FADS2 (fatty acid d5 and d6 desaturase) are key enzymes for synthesizing polyunsaturated fatty acids (PUFAs). Normally, aging is associated with increased serum levels of the pro-inflammatory PUFA, arachidonic acid. FADS1 can catalyze the conversion of the non-inflammatory PUFA 18:w6-linoleic acid to arachidonic acid (AA). An SNP that reduces FADS1 activity has been linked to a reduced age-associated increase in serum AA and reduced CVD-R. Higher serum levels AA are correlated with higher levels of IL-6 and TNFa [13]. Free fatty acid (FFA) levels in serum are correlated with insulin resistance and obesity [569]. FFAs are derived from lipid droplets through the hormone-sensitive lipase (HSL) and hormone-insensitive lipase (ATGL) catalyzed hydrolysis of TG. HSL is activated upon phosphorylation by protein kinase A (PKA), a cAMP-regulated kinase [570,571]. In the fasting state, adrenaline stimulates adenyl cyclase resulting in increased levels of cAMP and increased production of FFAs due to activation of HSL [572]. In the fed state, activation of phosphodiesterase3 (PDE3) by serine/threonine-specific protein kinase (AKT) (downstream of insulin receptor) reduces cAMP level, inhibiting HSL-catalyzed FFA formation and promoting lipid droplet enlargement. The prolonged caloric excess results in the accumulation of hypertrophied adipocytes, which secrete inflammatory cytokines that inhibit fatty acid incorporation into TG and increase the hydrolysis of TG to release FFAs. FFAs mediate insulin resistance by activating JNK1, IKkB, PKC-theta, mTORC1, and p70S6K kinases that inhibit IRS through serine phosphorylation. Mice with the knockout of these kinases have improved insulin sensitivity [573,574,575,576,577,578]. FFAs also mediate IR by activating TLR2 and TLR4 [579,580]. FFAs can activate TNFa signaling with consequent insulin resistance due to suppression of AKT function [577,581]. In addition to TNFa, hypertrophied adipocytes also produce MCP1, IL1b, and many other cytokines that attract macrophages.

Normally macrophages represent 5% of cells in adipose tissue. However, in inflamed adipose, up to 50% of cells may be macrophages [582]. Cytokine production by adipocytes and macrophages in adipose tissue is amplified through paracrine and autocrine signaling loops, exacerbating adipose inflammation. TNFa also increases cellular cAMP levels, thus activating HSL and increasing FFA production [570]. A major mechanism for lipid droplet dysfunction and FFA production is the inhibition of PPARg transcription by TNFa through activation of MAPK4, NFkB, and AP1 [583,584]. PPARg is a key adipocyte transcription factor that regulates lipid droplet homeostasis by controlling the expression of key lipid droplet-associated PAT-domain [585], CIDE-domain (CIDE-A and CIDE-C) and fat-induced transcript (FIT1, FIT2) [586] proteins that function to stabilize lipid droplets. Perilipin proteins coat the lipid droplets and protect them from lipolysis by HSL. MLDP, a lipid droplet scaffold protein, is inducible and provides protection during oxidative stress [587]. The FIT proteins play a role in lipid droplet formation and the CIDE proteins (cell death-inducing DNA fragmentation factor alpha-like effector) in their enlargement [588]. Formation of DAG results in activation of PKC and consequent activation of NFkB [46].

Summary: FFAs promote obesity, inflammation, and insulin resistance through activation of JNK, IKkB, PKC, NFκB, and TLR2/TLR4 pathways, leading to secretion of TNFα, IL-6, IL-1β, and MCP-1 from hypertrophied adipocytes. Macrophage infiltration of inflamed adipose tissue can reach 50% of cells, amplifying cytokine production through paracrine and autocrine loops, while TNFα-mediated inhibition of PPARγ disrupts lipid droplet homeostasis and further increases FFA release, creating a self-perpetuating cycle of inflammation and metabolic dysfunction.

## 17. Enzymes of Lipid Metabolism

Lipoprotein-associated lipase A2 (LPLA2), or PAF-AH, is a proinflammatory marker shown to predict cardiovascular events and is bound to LDL [589]. Higher LPLA2 activity, associated with smaller, more atherogenic LDL particles, increases CV risk by 1.85-fold. In a 2008 intervention trial, gemfibrozil increased HDL levels and decreased total cholesterol by inhibiting LPLA2 activity [589].

## 18. Genetic Markers

Since CVD-R is determined in most individuals through polygenic defects, it is hoped that a mechanistic understanding of the underlying causes of CVD will eventually lead to genetic means of accurate risk assignments for each person based on combinations of genetic characteristics. Classical genetics and, more recently, genome-wide association studies have identified many genes that can predispose CVD-R factors, including obesity, HTN, type-2 T2DM, metabolic syndrome, and coagulopathy. These findings are based primarily on the correlation of specific single-nucleotide polymorphisms in coding as well as non-coding regions of several genes known to be involved in the pathogenic mechanisms of CVD-R factors, and many others for which no mechanisms for predisposing CVD are evident. These associations are found in both univariate and multivariate analyses of CVD-R factors including obesity [590], CAC [591], HTN [592], T2DM [593], hyperlipidemia [594], and inflammation [595]. However, these polymorphisms, either alone or in combinations, are insufficient to yield clinically meaningful improvement of the predictive accuracy or precision of the Framingham Risk Score (FRS). Furthermore, the capacity of genetic tests to predict CVD-R is inherently limited by variations in individual exposures to environmental stress. Proteomics-based panels (e.g., SOMAscan, Olink platforms) [596,597] simultaneously measure a multitude of proteins translated in response to cardiovascular stress and have potential as novel CVD-associated protein signatures. Metabolomics approaches [598] identifying LPO-derived metabolite signatures, branched-chain amino acids, and trimethylamine N-oxide (TMAO) as CVD-R markers will need assessment with further prospective clinical trials.

Summary: GWAS have identified numerous SNPs associated with CVD-R factors including obesity, HTN, T2DM, and hyperlipidemia; these polymorphisms individually or in combination have not yielded clinically meaningful improvement over the Framingham Risk Score, largely due to variability in environmental stress exposures. Emerging proteomics (SOMAscan, Olink) and metabolomics (TMAO, branched-chain amino acids) platforms offer promise as novel CVD-R signatures but require further prospective clinical validation.

## 19. Conclusions

In conclusion, a variety of CVD biomarkers are presently commercially accessible and have clinical applications as diagnostic, prognostic, or predictive biomarkers. A number of these biomarkers need to undergo rigorous testing in order to determine their clinical applicability in a wide range of patients with atherosclerotic CVD and who have various comorbidities (Table 3). Before a biomarker is regularly used in clinical practice, it must first demonstrate its validity and clinical value across various patient populations. Any CVD biomarker should have the following desirable properties: simple measurement, ideally at point of care within a brief time period with sufficient precision and accuracy, and the showing of minimal intra-individual variability. Without duplicating any information that is already clinically accessible, a biomarker may be able to represent the pathophysiologic process of cardiac disease as well as offer significant information regarding prognosis and aid in guiding clinical decision-making. In order to show its superiority to conventional and other widely used biomarkers, biomarkers evaluating prognostic outcomes should report discrimination, calibration, and reclassification in patients by comparing statistical models with and without the biomarker. Since natriuretic peptides and cardiac troponins are cardiac-specific, they are more closely associated with cardiovascular diseases than assays for measuring inflammatory and oxidative stress, which are time-consuming, expensive, and frequently insensitive. However, the most significant factor contributing to these markers’ failure in multivariate analysis for cardiovascular risk or their poor diagnostic accuracy is that they are not cardiac-specific.

Although high platelet counts only considerably raise the risk of MI in myeloproliferative diseases, platelets drive coronary thrombus formation through degranulation-induced oxidative stress. Coagulation factors increase the risk of MI and stroke, especially fibrinogen (~2.3-fold IHD risk) and FGG mutations. With miR-128 and miR-195 directly suppressing NPRA expression, microRNAs (miR-622, -519, -499) have become promising heart failure biomarkers. RALBP1 (Rlip76) links oxidative stress detoxification to clathrin-dependent endocytosis, with knockout mice exhibiting extreme insulin sensitivity and resistance to obesity, suggesting potential as a unifying etiological CVD-R biomarker. The CRP is part of the Reynolds Risk Score and confers 1.3–1.5-fold CVD risk increase per unit. The JUPITER trial supports the use of statins in intermediate-risk adults with elevated CRP; however, the independent predictive improvement over FRS is still only 3–8%. IL-6 independently predicts MI mortality, with tocilizumab trials demonstrating reduced inflammation and myocardial damage, but clinical utility is limited by the short half-life and assay cost. TNFα shows dual cardioprotective/cardiotoxic properties with elevated TNFR1/TNFR2 linked to worse cardiovascular outcomes, though routine clinical adoption remains limited. Among lipokines, leptin shows modest CVD-R association (1.36–1.5-fold), while MCP-1 demonstrates the most recent promise through independent association with long-term cardiovascular mortality. Cell adhesion molecules do not predict MI mortality, though sVCAM-1 shows emerging potential for atrial fibrillation prognostication. Oxidized lipid biomarkers (F2 isoprostanes, PGF2α) serve as integrated markers of oxidative stress but do not improve prediction beyond FRS. Free fatty acids perpetuate inflammation through JNK, NFκB, and TLR pathways, with TNFα-mediated PPARγ inhibition disrupting lipid droplet homeostasis. LPLA2 predicts cardiovascular events with 1.85-fold increased risk.

Genetic biomarkers such as proteomics (SOMAscan, Olink) and metabolomics (TMAO, branched-chain amino acids) are at the forefront of being assessed for further usefulness, and they too require thorough evaluations to examine their relationships with CVD and for their increased benefit above conventional risk factors. The future of CVD therapy will probably be determined using biomarkers as surrogate endpoints for predictive and prognostic values in clinical trials. This will also provide opportunities to assess biomarkers as potential targets for drug delivery and development.

## Figures and Tables

**Table 1:** Routine diagnostic studies used at clinical level for cardiovascular risk determination.

Type of Study	Tests
Clinical Chemistry	Blood glucose
	BUN/Creatinine
	Potassium
	Magnesium
	Homocysteine
Serum Enzymes	γ-Glutamyltranspeptidase
	Creatine phosphokinase and isozymes
	Aspartate transaminase
	Glutamate transaminase
Serum Proteins	Troponins
	Hemoglobin A1C
	Complement proteins
	ICAM-1
Hormones	Insulin
	Thyroid hormone
Serum Lipids	TC
	LDL-C
	HDL-C
	Non-HDL-C
	LDL-C/HDL-C ratio
	ApoB
	Lysophosphatidyl choline
Coagulation Factors	Fibrinogen
	Factor VIII
	D-dimer
	Von Willebrand factor
	Tissue plasminogen activator (TPA)
Cytokines	CRP
	IL-6
	IL-10
	IL-13, -17, -18
	IL-27
	IL33
	TGF-beta
	TNFα
	Galectin-3
Adipokines	Leptin
	Adiponectin
Vasoactive Peptides	Natriuretic peptides (ANP, BNP)
	Angiotensin
	Bradykinin
Cardiac Peptides	Tropinin T and I
Redox Markers	Myeloperoxidase
	PON1
	Selenium
	Serum thiols
	Glutathione
	Cysteinylglycine
Acute-Phase Reactants	C-reactive protein
	Pentraxin-3
	Amyloid A
	Modified albumin
Eicosanoid Metabolites	6-keto prostaglanding F2α
	Lipid hydroperoxides
Non-invasive Imaging	Coronary calcification
	Carotid calcification
	Peripheral vascular disease
Genetic polymorphisms	CDKN2B, LPA, LPL, and APOC1
Invasive Imaging (Cardiac Cath)	Coronary stenosis
	Coronary thrombosis
	Coronary spasm

**Table 2:** Correlation of pathways involved with cardiovascular risk with Rlip in knockout mice.

Ingenuity Canonical Pathways	−log(*p*-Value)	zScore	Ratio
NRF2-mediated Oxidative Stress Response	1.44 × 10^1^	3.40	0.29
Cardiac Hypertrophy Signaling (Enhanced)	1.20 × 10^1^	3.67	0.21
RHOGDI Signaling	9.69 × 10^0^	2.20	0.26
STAT3 Pathway	8.12 × 10^0^	3.92	0.28
G-Protein Coupled Receptor Signaling	7.70 × 10^0^	2.03	0.17
Apelin Endothelial Signaling Pathway	6.09 × 10^0^	2.35	0.25
Noradrenaline and Adrenaline Degradation	5.74 × 10^0^	−2.67	0.41
Fatty Acid α-oxidation	5.69 × 10^0^	−3.16	0.52
Ephrin B Signaling	5.63 × 10^0^	3.87	0.31
TGF-β Signaling	4.92 × 10^0^	2.84	0.26
Apelin Adipocyte Signaling Pathway	4.85 × 10^0^	3.58	0.26
Acute-Phase Response Signaling	4.84 × 10^0^	3.41	0.21
Coagulation System	4.57 × 10^0^	3.05	0.37
Superpathway of Cholesterol Biosynthesis	4.04 × 10^0^	2.11	0.38
Role of NFAT in Cardiac Hypertrophy	4.00 × 10^0^	2.12	0.19
Endothelin-1 Signaling	3.76 × 10^0^	2.74	0.19
Superpathway of D-myo-inositol (1,4,5)-trisphosphate Metabolism	3.69 × 10^0^	3.00	0.41
FGF Signaling	3.35 × 10^0^	3.15	0.23
α-Adrenergic Signaling	3.18 × 10^0^	2.11	0.21
D-myo-inositol (1,3,4)-trisphosphate Biosynthesis	3.00 × 10^0^	2.65	0.41
Cholesterol Biosynthesis I	2.94 × 10^0^	2.45	0.46
Cholesterol Biosynthesis II (via 24,25-dihydrolanosterol)	2.94 × 10^0^	2.45	0.46
Cholesterol Biosynthesis III (via Desmosterol)	2.94 × 10^0^	2.45	0.46
Glutathione-mediated Detoxification	1.92 × 10^0^	2.65	0.24
Regulation Of The Epithelial–Mesenchymal Transition In Development Pathway	1.77 × 10^0^	2.32	0.18
Gαi Signaling	1.73 × 10^0^	2.32	0.16
Gα12/13 Signaling	1.71 × 10^0^	3.41	0.17
D-myo-inositol (1,4,5)-trisphosphate Degradation	1.11 × 10^0^	2.00	0.25
PDGF Signaling	5.48 × 10^−1^	2.11	0.13
CSDE1 Signaling Pathway	4.36 × 10^−1^	−2.45	0.13
Oxidative Phosphorylation	4.28 × 10^−1^	−3.46	0.12
VEGF Family Ligand-Receptor Interactions	0.00 × 10^0^	2.24	0.06
NF-KB Signaling	0.00 × 10^0^	2.19	0.06
Netrin Signaling	0.00 × 10^0^	−2.00	0.08

**Table 3:** Descriptive summary of all cardiovascular biomarkers with their strength, limitations, prognostic significance and interpretation in context of comorbidities.

Biomarker	Strengths for CVD Risk Prediction	Limitations in Clinical Usage	Prognostication Value	Variation with Health Comorbidities
**Troponin**	Gold standard for myocardial injury Highly specific immunoassays (cTnI 50% homology to other isoforms) High-sensitivity assays detect minute myocardial damage Negative tests permit safe ER discharge Earlier detection than CK-MB	Marker of injury, not etiology Baseline varies by gender and age (biphasic: decreases in teens, stable in adulthood, rises after age 60)	30-day mortality predictor and 2-year risk stratification	**CKD:** Predicts risk regardless of renal function **Diabetes:** Reliable predictor of CV death, MI, stroke in T2DM with stable IHD **Inflammatory:** Additive risk with CRP
**Natriuretic Peptides (BNP/NT-proBNP)**	Most widely used diagnostic biomarkers for CHF and BNP correlates with CHF severity Recommended for diagnosis, monitoring, and prognosis of HF Useful for surgical CV risk evaluation in patients >55 years	Must consider age and body mass ANP has short half-life (proteolytic sensitivity) Complex proteolytic processing yields multiple peptides with overlapping effects	Powerful short-term prognosis markers Treatment response predictor BNP dynamic range correlates with CHF severity	**Diabetes:** Lower NP levels, defective NP receptor signaling, increased NPR3 clearance in T2DM **Inflammatory:** Modulates TNFα, IL-6, TGFB, NFKB expression
**Blood Cells**	Platelets key in coronary thrombus formation Antiplatelet agents universally reduce CVD risk Platelet degranulation generates oxidative stress (lipid hydroperoxides, eicosanoids)	Elevated platelet count alone does not predict MI Thrombotic risk may not parallel platelet count	WBC count may have prognostic utility in myeloproliferative disorders Intrinsic platelet abnormalities more relevant than absolute counts	**Inflammatory:** Reactive thrombocytosis occurs with inflammation but does not significantly increase MI risk Inflammatory cytokines associated with platelet abnormalities
**Coagulation Factors (Fibrinogen)**	Fibrinogen strongly correlated with CVD risk	Fibrinogen is covariate of most CVD risk factors Elevated post-MI as acute-phase reactant Gene polymorphism impact unclear due to CRP/lipid gene interactions	Fibrinogen: 2.3–5× IHD risk D-dimer superior to CRP/IL-6 for acute coronary events	**Diabetes:** Fibrinogen elevated in T2DM, PAI-1 1.5× CVD risk in T2DM **Inflammatory:** Fibrinogen rises with inflammation, IL-6 promotes synthesis **Liver:** Synthesized in hepatocytes
**MicroRNAs**	Potential diagnostic, prognostic, severity markers miR-622, -519, -499 related to CHF etiology	Described as “potential” markers suggesting ongoing validation Translation to clinical practice requires further development	Let-7i-5p, miR-223-5p, miR-423-5p, miR-21, miR-1306-5p, miR-122 correlate with prognosis and COVID-19 survival miR-22-3p assesses CHF severity	**COVID-19:** Multiple miRNAs correlate with prognosis and survival
**Ralbp1/Rlip76**	Elevated Rlip in metabolic syndrome vs. normal individuals Stress-inducible Links CDE with lipid hydroperoxide detoxification	Awaits completion of cardiovascular characterization in knockout mice and human clinical studies Current evidence primarily from animal models and transcriptomic analyses	Potential etiological biomarker but clinical validation needed	**Diabetes:** Knockout mice extremely insulin sensitive, hypoglycemic **Metabolic Syndrome:** Elevated Rlip in metabolic syndrome **Obesity:** Knockout mice resistant to diet-induced obesity
**C-Reactive Protein (CRP)**	Most investigated CVD biomarker globally Standardized hsCRP assays Incorporated into Reynolds Risk Score (reclassifies 50% women)	Cannot identify specific CV etiology Improves Framingham Risk Score by only 3–8% May be consequence rather than cause of CVD	2× coronary event risk (>2.4 mg/L) 1.3–1.5× CVD risk (meta-analysis) 2× stroke risk independent of smoking, BP, HDL-C, DM	**Metabolic Syndrome:** Inhibits endothelial NO, activates monocytes, predictive value beyond conventional criteria may be small **Inflammatory:** Acute-phase reactant, rises with inflammation
**IL-6**	Independent predictor of MI mortality Correlates directly with CVD; early indicator (rises before temperature/acute-phase proteins)	Higher cost than other biomarkers, short half-life, difficult to assay Elevated in comorbidities like sepsis and other factors like age, diabetes, exercise alter levels	Independent MI mortality predictor Higher IL-6 correlates with poor outcomes in unstable angina, poor LVF, higher restenosis rates Useful for treatment efficacy assessment	**Diabetes:** Alters IL-6 concentrations **Sepsis:** May reach levels reach 1600 pg/mL **Anemia:** IL-6-hepcidin axis causes hypoferremia in chronic inflammation
**TNF Alpha**	Implicated in atherosclerosis via lipid metabolism, endothelial activation, vascular inflammation	Limited clinical application from lengthy assay incubations Dual nature (cardioprotective for failing/cardiotoxic for healthy myocardium) complicates interpretation	TNFR1/TNFR2 linked to higher CV events and mortality, but minimal enhancement of risk prediction	**Diabetes:** Linked to increased CVD risk, causes hyperglycemia **Obesity:** Increased CVD risk **Inflammatory/Autoimmune:** Higher CVD risk in RA, psoriatic arthritis **CKD:** Causes acute renal tubular necrosis
**Leptin**	Correlates with BMI, insulin, C-peptide, sTNFR1/2	Not routine due to cost and few prospective studies No account for leptin resistance in humans and not correlated with CVD risk in large European study (unlike CRP, IL-6)	Marginally significant, limiting utility Meta-analysis showed 1.36–1.5 times CVD risk (men/women)	**Obesity:** High levels, directly correlated with BMI **Diabetes:** Correlates with HgbA1C (BMI > 25 only) **Metabolic Syndrome:** Correlates with insulin, C-peptide, inflammatory markers
**Adiponectin**	More associated with DM and visceral adiposity (inversely)	Not used routinely Has high levels in diabetics and chronic inflammatory diseases, making it a poor biomarker	No specific numerical prognostic data Independent role in DM/insulin resistance unlikely	**Obesity:** Low levels with visceral adiposity **Diabetes:** Low levels associated with DM risk but high levels occur in diabetics **Chronic Inflammatory:** High levels noted
**RBP4**	Higher in men, correlates with BMI, insulin resistance measures Reduced with bariatric surgery weight loss	New biomarker needing additional prospective studies Inconsistent relationship with obesity/insulin resistance across studies	Limited evidence on prognostication	**Diabetes:** Increased in T2DM (inconsistent) **Obesity:** Correlates with BMI **CKD:** Renal dysfunction affects levels
**Resistin**	Correlates with coronary atherosclerosis severity, CHF and CAD presence	Limited literature Hypothesis for association with CVD risk limited	Predicts coronary restenosis post-MI in diabetics (no specific numerical data)	**Obesity:** High levels, correlates with BMI **Diabetes:** Predicts restenosis post-MI in DM **Inflammatory:** Correlates with CRP, IL-6, TNF
**MCP-1**	Correlates with MI/atherogenesis risk factors (age, smoking, TG, BMI, waist)	Additional prospective studies needed	Higher levels linked to long-term CV mortality (no specific numerical data)	**Obesity:** Association depends on ethnicity, gender, menopausal status
**P-Selectin**	Associated with metabolic syndrome risk independent of other Framingham Risk Score factors (unlike CRP, IL-6, MCP1, TNF)	Weak association with CVD risk prediction	Limited prognostication value	**Metabolic Syndrome:** Weakly associated (1.06-fold)
**Lipocalin**	Correlates with obesity, TG, insulin resistance Useful for PAD risk stratification	Limited literature	May be useful for PAD risk stratification	**Diabetes/Insulin Resistance:** Correlates to certain extent **Dyslipidemia:** Correlates with TG, inversely with HDL-C
**TGF Beta**	High levels marginally associated with higher DM risk but convincing cell/animal evidence	Clinical associations not convincing	Limited CVD prognostic data	**Diabetes:** Marginally significant higher DM risk
**Chemerin**	Recent prospective cohort: significant independent relationship with CVD	Poor predictor of CVD risk in some studies Requires additional prospective studies	Significant independent CVD relationship (no specific numerical data)	**Obesity:** Increased; weight loss reduces **Diabetes:** Large positive T2DM correlation (partially BMI-attributable) **Metabolic Syndrome:** Increased in early metabolic syndrome
**Other Cytokines/Lipokines**	Omentin correlates with adiponectin IL-1RA independently relates obesity to insulin resistance	SFRP5: Inconsistent results, very low expression in human fat Visfatin: Inconsistent correlations with atherosclerosis/DM Apelin: Weak DM correlation NF1: Narrow weight range, men only	Variable	**Diabetes:** Low omentin may predict post-MI complications, apelin increased (weak), vaspin associated with T2DM **Obesity:** Vaspin correlates with BMI
**Cell Adhesion Molecules**	sVCAM-1 may be biomarker for AF, POAF, CV death in CABG patients with CAD as endothelial cells are primary source of ICAM/VCAM	MI mortality not predicted by sICAM, sVCAM-1, P-selectin More development needed in the biomarkers	sVCAM-1 may predict CV death in CABG patients (validation needed)	**Atrial Fibrillation:** sVCAM-1 may be biomarker for AF and POAF
**Oxidized Lipids**	9-HETE and F2-isoprostanes strongly associated with angiographic CAD F2-isoprostanes established lipid peroxidation biomarkers PGF2a: Integrated marker of oxidative stress, inflammation, BMI, age PON1 activity correlates with CVD risk	Does not improve ROC beyond traditional Framingham Risk Score factors Cause–effect unclear	Strong angiographic CAD association but no multivariate model improvement	**Obesity:** Weight gain increases PGF2a, low PON activity **Diabetes:** Low PON activity in T2DM, PGF2a increased in insulin resistance **CKD:** Low PON activity **Liver:** Low PON activity in hepatic cirrhosis
**Free Fatty Acids**	Key role in obesity, inflammation, oxidative stress Activate TNF, MCP-1, IL-6 secretion Acutely induce insulin resistance Correlate with insulin resistance and obesity	No specific data on clinical biomarker utility Pathophysiology established but standalone biomarker utility not addressed	Specific prognostic data limited	**Obesity:** Elevated FFA, activate inflammatory cytokines **Insulin Resistance:** Acutely induce via multiple kinase pathways **Inflammatory:** Activate TNF, MCP-1, IL-6, up to 50% macrophages in inflamed adipose (vs. 5% normal)
**Enzymes of Lipid Metabolism (LPLA2)**	LPLA2/PAF-AH predicts CV events; bound to LDL Higher activity associated with smaller, more atherogenic LDL	Inconclusive literature on limitation	1.85-fold increased CV risk with higher LPLA2 activity	**Dyslipidemia:** Associated with smaller, more atherogenic LDL particles
**Genetic Markers**	Genome-wide studies identified genes predisposing CVD risk factors (obesity, HTN, T2DM, metabolic syndrome, coagulopathy)	Inherently limited by individual environmental stress exposure variations	Identify predisposition to CVD risk factors but do not improve Framingham Risk Score prediction	**Obesity, HTN, T2DM, Metabolic Syndrome, Coagulopathy, Hyperlipidemia, Inflammation:** Genetic associations identified but clinical predictive value limited by environmental factors

## Data Availability

No new data were created or analyzed in this study. Data sharing is not applicable to this article.

## Abbreviations

AA	Arachidonic Acid
ADP	Adenosine Diphosphate
AF	Atrial Fibrillation
AKT	Serine/Threonine-Specific Protein Kinase (Protein Kinase B)
ALT	Alanine Transaminase
ANP	Atrial Natriuretic Peptide
AST	Aspartate Transaminase
ATP	Adenosine Triphosphate
BMI	Body Mass Index
BNP	B-type Natriuretic Peptide
BP	Blood Pressure
CAD	Coronary Artery Disease
CABG	Coronary Artery Bypass Grafting
cAMP	Cyclic Adenosine Monophosphate
CBC	Complete Blood Count
CBD	Cytokine Binding Domain
CDE	Clathrin-Dependent Endocytosis
cGMP	Cyclic Guanosine Monophosphate
CHF	Congestive Heart Failure
CK	Creatine Kinase
CKD	Chronic Kidney Disease
CK-MB	Creatine Kinase-MB Isoform
CMP	Comprehensive Metabolic Panel
CNP	C-type Natriuretic Peptide
CNS	Central Nervous System
CPK	Creatine Phosphokinase
CRP	C-Reactive Protein
cTnI	Cardiac Troponin I
cTnT	Cardiac Troponin T
CV	Cardiovascular
CVD	Cardiovascular Disease
CVD-R	Cardiovascular Disease Risk
DAG	Diacylglycerol
DAMPs	Damage-Associated Molecular Patterns
DM	Diabetes Mellitus
DPP4	Dipeptidyl Peptidase-4
EGF	Epidermal Growth Factor
EKG	Electrocardiogram
ELISA	Enzyme-Linked Immunosorbent Assay
FFA	Free Fatty Acid
FGA	Fibrinogen Alpha Gene
FGB	Fibrinogen Beta Gene
FGF	Fibroblast Growth Factor
FGF21	Fibroblast Growth Factor 21
FRS	Framingham Risk Score
GWAS	Genome-Wide Association Studies
HDL	High-Density Lipoprotein
HDL-C	High-Density Lipoprotein Cholesterol
HF	Heart Failure
HSL	Hormone-Sensitive Lipase
hsCRP	High-Sensitivity C-Reactive Protein
HTN	Hypertension
ICAM 1	Intercellular Adhesion Molecule-1
IGF1	Insulin-Like Growth Factor 1
IHD	Ischemic Heart Disease
IL	Interleukin
IRS1	Insulin Receptor Substrate 1
JNK	Jun N-Terminal Kinase
LANP	Long-Acting Natriuretic Peptide
LDH	Lactate Dehydrogenase
LDL	Low-Density Lipoprotein
LOOH	Lipid Hydroperoxide
LPO	Lipid Peroxidation
LVF	Left Ventricular Function
MCP-1	Monocyte Chemoattractant Protein-1
MI	Myocardial Infarction
MIF	Macrophage Migration Inhibitory Factor
MINOCA	Myocardial Infarction with Non-Obstructive Coronary Arteries
mTORC1	Mechanistic Target of Rapamycin Complex 1
nCRP	Native C-Reactive Protein
mCRP	Monomeric C-Reactive Protein
NF1	Neurofibromatosis 1 gene
NFkB	Nuclear Factor Kappa-B
NK	Natural Killer (cells)
NO	Nitric Oxide
NP	Natriuretic Peptide
NPP	Natriuretic Peptide Precursor
NPR-3	Natriuretic Peptide Receptor 3
NSTEMI	Non-ST-Elevation Myocardial Infarction
NT-proBNP	N-Terminal Pro-B-Type Natriuretic Peptide
OPG	Osteoprotegerin
OX-S	Oxidative Stress
PAD	Peripheral Artery Disease
PAF-AH	Platelet-Activating Factor Acetylhydrolase
PAI-1	Plasminogen Activator Inhibitor-1
PCSK-9	Proprotein Convertase Subtilisin/Kexin Type 9
PDE3	Phosphodiesterase 3
PDGF	Platelet-Derived Growth Factor
PGF2a	Prostaglandin F2 Alpha
PKA	Protein Kinase A
PKC	Protein Kinase C
POAF	Postoperative Atrial Fibrillation
PON1	Paraoxonase-1
PPARg	Peroxisome Proliferator-Activated Receptor Gamma
PRKCA	Protein Kinase C Alpha
PRRs	Pathogen-Recognition Receptors
PUFA	Polyunsaturated Fatty Acid
qHTS	Quantitative High-Throughput Screening
RALBP1	Ral-Binding Protein 1
RBP4	Retinol-Binding Protein 4
RGS2	Regulator of G-Protein Signaling 2
Rlip76	Ral-Interacting Protein of 76 kDa
ROC	Receiver Operating Curve
ROS	Reactive Oxygen Species
SAP	Serum Amyloid P Component
SAT	Subcutaneous Adipose Tissue
SFRP5	Secreted Frizzled-Related Protein 5
SGOT	Serum Glutamate-Oxaloacetate Transaminase
SGPT	Serum Glutamate-Pyruvate Transaminase
sICAM	Soluble Intercellular Adhesion Molecule
sIL-6R	Soluble Interleukin-6 Receptor
SNP(s)	Single Nucleotide Polymorphism(s)
SOCS	Suppressor of Cytokine Signaling
Sp1	Specificity Protein 1
SREBP-2	Sterol Regulatory Element-Binding Protein-2
sTNFR1	Soluble Tumor Necrosis Factor Receptor-1
sTNFR2	Soluble Tumor Necrosis Factor Receptor-2
sVCAM1	Soluble Vascular Cell Adhesion Molecule-1
T2DM	Type 2 Diabetes Mellitus
TACI	Transmembrane Activator and CAML Interactor
TC	Total Cholesterol
TG	Triglyceride
TGF-beta	Transforming Growth Factor Beta
TLR	Toll-Like Receptor
TMAO	Trimethylamine N-Oxide
Tn	Troponin
TNFa	Tumor Necrosis Factor Alpha
TNF-R1	Tumor Necrosis Factor Receptor 1
TPM	Tropomyosin
TRAIL	TNF-Related Apoptosis-Inducing Ligand
U/A	Urinalysis
UV	Ultraviolet
VAT	Visceral Adipose Tissue
VCAM	Vascular Cell Adhesion Molecule
VD	Vessel Dilator
VLDL	Very Low-Density Lipoprotein
VSMCs	Vascular Smooth Muscle Cells
vWF	Von Willebrand Factor
WNT	Wingless-Related Integration Site
WSXWS	Tryptophan-Serine-X-Tryptophan-Serine (motif)
9-HETE	9-Hydroxyeicosatetraenoic Acid
